# Engineering anisotropic tissues: from structured scaffolds to magnetic actuation

**DOI:** 10.1016/j.mtbio.2026.103191

**Published:** 2026-05-02

**Authors:** Noam Demri, Stéphanie Descroix, Claire Wilhelm

**Affiliations:** Laboratoire Physique des Cellules et Cancer, PCC, CNRS UMR168, Institut Curie, Sorbonne University, PSL University, Paris, 75005, France

**Keywords:** Anisotropy, Tissue engineering, Biomaterials, Morphogenesis, Biomechanics, Magnetic forces

## Abstract

Tissue engineering emerged in the late 20th century to replicate human tissues *in vitro* for biomedical applications. Early concepts relied on culturing cells within three-dimensional scaffolds to reproduce *in vivo* architecture. However, native tissues are not only three-dimensional but also structurally complex, heterogeneous, and often anisotropic - skeletal muscle being an archetypal example. These anisotropic features are not merely structural, as they critically influence tissue mechanics and function. Replicating such multiscale structural and mechanical complexity is therefore critical to engineer physiologically relevant tissue models. After outlining the diversity and functional significance of anisotropic tissues *in vivo*, this review examines current material- and fabrication-based strategies for anisotropic tissue engineering. Approaches range from surface-engineered 2D substrates and architected polymeric scaffolds to hydrogel-based three-dimensional bioprinting, where micro- and nano-scale control over material properties enables guided cell alignment. In addition, emerging techniques exploit external forces, such as electrical or acoustofluidic stimuli, to induce structural micro-features, while others leverage the intrinsic self-organization capacity of cells. Among these externally driven approaches, magnetic-based strategies are particularly promising due to their ability to provide remote, spatially precise, and dynamically tunable control over tissue organization, both at the microscopic and macroscopic scales. This review highlights their capacity to generate anisotropic architectures unattainable by conventional methods and discusses the key challenges that must be addressed to establish magnetic-based approaches as a promising emerging strategy to expand the design space for engineering functional, anisotropic tissues.

## Graphical abstract

**Figure:**
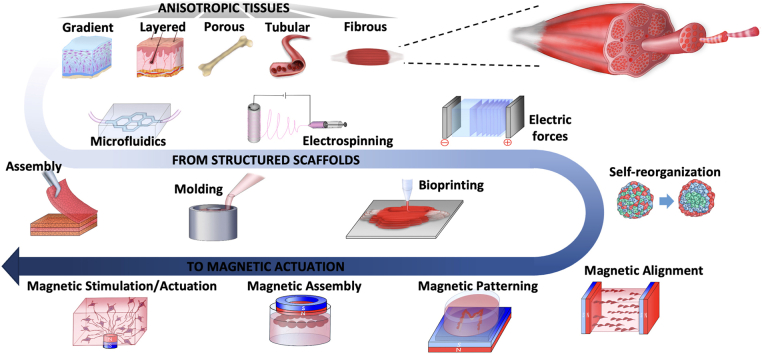

## 1 Introduction

Since tissue engineering was first introduced by Drs. J. Vacanti and R. Langer in the late 1980's [[1], [2], [3]], it has become more and more evident that two-dimensional (2D) cell culture is not sufficient to fully recapitulate the complexity of native tissues. Not only has three-dimensional (3D) cell culture advanced our understanding of *in vivo* biological processes [4], but it also offers significant potential for biomedical applications, notably new models for drug screening and potential grafts [5]. Although 3D culture can already achieve far more elaborate structures than 2D culture, it has not yet recapitulated the full complexity of *in vivo* tissues [6,7]. Unlike isotropic homogenous cell seeded scaffolds, *in vivo* organs have intricate geometries and many present key anisotropic features. Indeed, multiple tissues in the human body have structural and functional properties, such as stiffness, that vary depending on the direction of measurement [8]. Skeletal muscle tissue is a prime example of anisotropy in native tissues, as it presents a strong direction of alignment at every scale, from the whole organ to myofibrils, all oriented in the same direction [9]. This marked fibrous anisotropy links geometry to function, as this alignment optimizes the coordinated and uniform contraction of all functional units of the tissue for force generation. Indeed, anisotropy is not merely a structural feature, as it is crucial for the functions of all anisotropic tissues and organs. Replicating such complex architectures *in vitro* has therefore emerged as the next logical step in tissue engineering to better recapitulate native anatomy [[10], [11], [12], [13]].

Starting with the use of anisotropic 2D substrates [14], anisotropic tissue engineering has progressively evolved towards 3D approaches such as modular assembly or moulding, eventually leading to the emergence of organ-on-chips technologies [[15], [16], [17], [18], [19], [20], [21], [22]]. The development of electrospinning [23] and bioprinting [24,25] has enabled the creation of even more complex anisotropic geometries. However, despite these advances, most conventional approaches remain limited by their reliance on predefined scaffold architectures, which restrict dynamic control over tissue organization and make it difficult to modulate structures deep within thick, 3D constructs. In particular, achieving remote, spatiotemporal control of anisotropy and recreating dynamic tissue remodeling processes remain significant challenges.

As a result, new approaches have begun to use strategies for remote control, such as acoustic or magnetic forces, as tools for tissue engineering and stimulation. Based on the use of magnetic nanoparticles, originally used for imaging purposes [26], magnetic tissue engineering started with the generation of cellular sheets [27]. This technique gradually increased in complexity and demonstrated an ability to create intricate anisotropic architectures [[28], [29], [30], [31]], while at the same time enabling remote stimulation [32,33]. By enabling contactless, reversible, and spatially controlled actuation, magnetic approaches offer a promising route to overcome several of the key limitations of conventional scaffold-based strategies. Magnetic bioprinting, possibly combined with other current techniques, is emerging as a powerful tool with the potential to significantly advance anisotropic tissue engineering.

After providing an overview of the various anisotropic tissues in the human body, this review surveys the different strategies aiming to replicate anisotropy *in vitro*, including approaches harnessing the cells innate self-organizing properties. These approaches are presented as the foundational strategies that have enabled controlled anisotropy but also highlight remaining limitations. Importantly, magnetic-based techniques will be introduced as a promising avenue for anisotropic tissue engineering.

## 2 Anisotropic tissues in the human body: structure, mechanics, and function

a. Defining and assessing anisotropy in living tissues

The hallmark of an anisotropic material is a variation of its properties depending on the direction in which they are measured. Importantly, anisotropy does not necessarily imply spatial heterogeneity, as a material can be homogeneous while exhibiting direction-dependent properties. Anisotropy should also not be confused with geometrical alignment, which represents only one possible manifestation. For instance, wood, with an elastic modulus about 10 times higher in the longitudinal direction than in the radial one, is a common example of anisotropic material. Such materials contrast with isotropic materials, whose properties are uniform, independently of the direction, such as glass or plastic.

In living tissues, anisotropy arises across multiple scales, from the elongated geometry of certain organs down to the polarization and alignment cells, or even organization of extracellular matrix components. These structural features can usually be evaluated by imaging techniques such as histology, fluorescence, polarized light and second harmonic generation microscopy, from which orientation distributions or alignment indexes can be extracted. These imaging techniques can also evaluate anisotropic variations in the composition of a tissue, such as changes in the orientation or the nature of cells or matrix elements. Beyond organization, anisotropy also manifests in functional properties, including directional mechanical behavior, transport, and conductivity. These properties can be assessed through directional mechanical testing, tracer-based transport assays, or electrical measurements. Quantitative descriptors such as alignment indices, orientation distributions, directional modulus ratios, and permeability coefficients provide a framework for objectively comparing anisotropic features across native and engineered tissues.

**Fig. 1:**
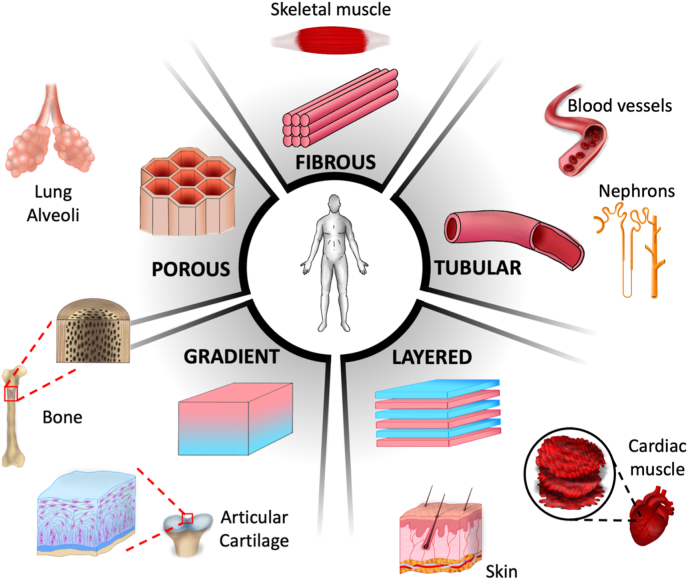
**Main classes of anisotropic tissues*****in vivo*****.**

Based on these criteria, anisotropic tissues can be classified under multiple architectural categories. For instance, skeletal muscle, with its aligned myofibers, falls under what will be called **fibrous** anisotropy. Some tissues or organs, with a more duct like geometry, fit in the **tubular or hollow** geometry whereas others display a **layered** structure. These three categories, first introduced in a previous review [8], can also be complemented with **gradient** and **honeycomb or porous** anisotropic architectures (Fig. 1). As will be discussed, several tissues possess attributes from more than one of these categories, thereby combining their respective advantages, and optimizing their material properties with regards to their anisotropic functionality.

b. Fibrous tissues

**Fig. 2:**
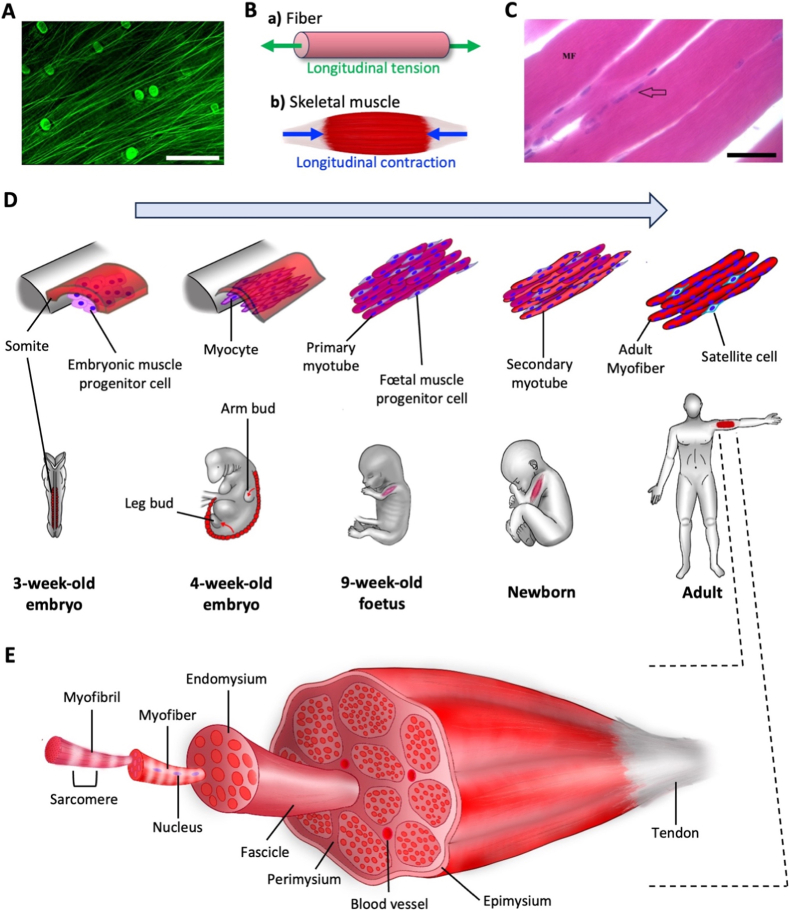
**Fibrous anisotropic tissue: the most common anisotropy in the human body.** (A) Confocal microscopy of aligned elastin fibers in the superficial zone of articular cartilage of kangaroo femoral condyle (Scale bar = 50 μm). Reproduced from Ref. [34]. (B) Schematics of the main stresses at play in fibrous tissues, either (a) exerted on a fiber or (b) produced by skeletal muscle tissue. Green arrows represent the stresses applied from the surrounding environment on the tissues and blue arrows represent the stresses due to muscle contractions. (C) Longitudinal histological sections of gastrocnemius muscle fibers (MF) with multiple nuclei (arrow) (Scale bar = 50 μm). Reproduced from Ref. [35]. Diagrams of (D) myogenesis at every stage of human development, leading to the formation of (E) the hierarchical aligned structure of adult skeletal muscle. (For interpretation of the references to colour in this figure legend, the reader is referred to the Web version of this article.)

Fibrous anisotropic tissues encompass all tissues composed of an assembly of fiber-like structures, organized so that they all share a similar orientation (Fig. 2A). This alignment gives the tissue preferential properties along that direction and directly contributes to their mechanical function.

Fibrous materials are present throughout the body, in extracellular matrices and basement membranes. They are composed of an entangled network of crosslinked filamentous proteins, classified as either collagen, reticular or elastic fibers [36]. Organized in 1 to 100 μm-thick bundles of fibrils, each between 20 and 500 nm in diameter and spanning up to several tens of millimeters [37], collagen fibers are mostly present in connective tissues such as tendons, ligaments, bone or cartilage [38]. Reticular and elastic fibers are smaller, with diameters below 2 μm and fibrils no more than 40 nm wide [39]. Although these different fibers are often arranged in an isotropic mesh, they are individually anisotropic due to both their elongated geometry and their mechanical properties. For instance, type I collagen fibrils and fibers in PBS exhibit a Young's modulus – i.e. the materials' resistance to elastic deformation - on the order of 100 MPa along the longitudinal direction (Fig. 2B), whereas their shear modulus - the resistance to sliding between layers in a material - is a hundred times smaller in the perpendicular direction [40,41]. This gives collagen fibers much higher resistance under tensile stress - the pulling force per unit area - although they may buckle when compressed [41].

When all these fibers align within an extracellular matrix, it leads to the formation of fibrous anisotropic tissues with much higher resistance to stress in the alignment direction. This can be observed in skin, where a dense network of collagen fibers aligns along Langer's lines, natural orientation patterns in the dermis. Consequently, the Young's modulus of the skin is ten times higher when measured parallel to these lines than perpendicular to them [42]. The impact of fiber orientation is also very visible in articular cartilage, where collagen fibers transition from aligned to isotropic, leading to a decrease in the Young's modulus parallel to the initial alignment. On the other hand, the Young's modulus in the perpendicular direction can be 10 to 50 times smaller than in the parallel direction in the aligned parts of the tissue [43,44]. Therefore, such fibrous alignment is particularly interesting for tissues meant to withstand uniaxial loads. This applies to tendons, linking muscles to bones, and ligaments, stabilizing joints or bones together. They share a similar structure [45] and are mostly composed of parallel collagen fibers, resulting in a Young's modulus two orders of magnitude higher in the parallel direction than the transverse one [46,47]. Their high tensile strength - the maximum pulling stress before failure - of about 100 MPa enables proper force transmission without failure [48], while their lower resistance to transverse deformations allows for greater flexibility and range of motion in the musculoskeletal system.

Alignment not only optimizes force transmission but also enhances force generation in skeletal muscle tissue. Unlike tendons and ligaments which exhibit a low cell density, skeletal muscle tissue draws most of its anisotropy not from collagen fibers, but from the densely packed muscle cells, the muscle fibers or myofibers (Fig. 2C), all aligned in the direction of contraction of the muscle (Fig. 2B). This anisotropy originates during early myogenesis (Fig. 2D). In somites, arises the myotome layer which will eventually lead to the formation of muscles [49]. Following a cascade of transcription factors [50], mononucleated muscle precursor cells differentiate into myocytes, elongate and align [51], displaying the first mechanism at the origin of the fibrous anisotropy of skeletal muscles [52]. This alignment enables the fusion of mononucleated myocytes into multinucleated myotubes, by favoring recognition and adhesion between consecutive cells [53]. This development results in skeletal muscle tissue composed, at every scale, of highly aligned fiber-like structures (Fig. 2E). At the organ level, skeletal muscles have an elongated structure, usually with a high aspect ratio which can reach 10 to 20 for the sartorius muscle [54]. Each muscle is connected on either end to tendons, transmitting the forces generated by muscles to bones in most cases. With a length of at least a few centimeters long [55] and a width between 40 and 100 μm [56], skeletal muscle cells not only have one of the highest aspect ratios but are also the largest cells in the human body [55]. The center of the cell is packed with thousands of myofibrils [57], aligned in the same direction as the cells . Myofibrils are characterized by a pattern of striations which represent the series of successive sarcomeres, the contractile units of the cells, each 2 to 3 μm-long [55]. These structures are composed of overlapping filaments of myosin and actin whose relative sliding produces contraction [[58], [59], [60]]. This molecular sliding is transmitted across multiple levels of organization, resulting in coordinated contraction along a single axis. Individual sarcomeres can generate stresses of the order of 10 to 100 nN μm^−2^ [61,62], resulting in whole-muscle forces up to 1 kN for large muscles like the quadriceps [63,64].

While skeletal muscle represents the most prominent type of anisotropic fibrous tissue, cardiac and smooth muscles also display some fibrous like characteristics. Their cells are much smaller [65], but still showcase a preferred alignment and elongation in the direction of contractions, as well as a main orientation of contractile filaments. In both cases they form layers of cells with a specific fibrous anisotropy, and the combination of different layer orientations leads to a layered anisotropy, as well as tubular anisotropy for smooth muscle cells.

In addition to muscle cells, neurons exhibit an aligned fibrous geometry, with 0.1 to 10 μm-thick [66] axons often extending across several centimeters, and even 1 m in the spinal cord [67]. These axons can share the same path, forming anisotropic fibrous tissues. While this naturally enhances mechanical performance in the fiber direction [68], the main function of this alignment is here to optimize and coordinate the transport of information.

Overall, these examples highlight that engineering fibrous anisotropic tissues requires reproducing not only cellular or matrix alignment, but also sufficient mechanical anisotropy and hierarchical organization across scales.

c. Tubular tissues

After the fibrous geometry, the next most prominent anisotropic architecture in the body is the tubular one, as many vessels and conduits of all shapes and sizes run through the body.

**Fig. 3:**
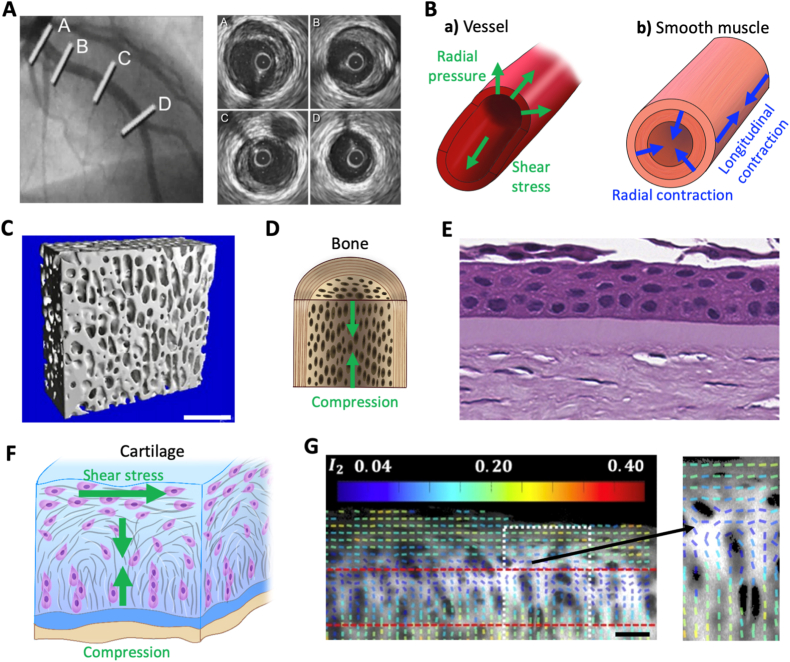
**Anisotropy in native tissues beyond fibrous alignment.** (A) Angiogram (left) and intravascular ultrasound imaging (right) of the left anterior descending artery (with a diameter of a few millimeters). Reproduced from Ref. [69]. (B) Schematics of the main stresses at play in tubular tissues, either (a) exerted on a vessel or (b) produced by smooth muscle tissue. (C) Microtomography imaging of trabecular bone (Scale bar = 2 mm). Reproduced from Ref. [70]. (D) Schematic of the compression stresses at play in bone tissue. (E) Histological section of the first layers of a human cornea: the epithelium, the Bowman's layer (approximately 10 μm) and the stroma. Reproduced from Ref. [71]. (F) Schematic of the main stresses at play in cartilage. (G) Map of the orientation and alignment (I_2_) of collagen fibrils in a cartilage-bone explant (Scale bar = 50 μm). Reproduced from Ref. [72].

The most typical tubular tissues are the blood vessels (Fig. 3A). From the 3 cm-wide aorta to less than 10 μm-wide capillaries [73], the vascular system exhibits a wide range of tubular geometries. Having a blood flow that can reach up to a few meters per second during exercise [74], these tissues have to withstand shear stresses - the parallel force from fluid flow over a surface - ranging from 10 to 100 dyne/cm^2^ (Fig. 3B). The endothelial cells lining the lumen of blood vessels are meant to withstand such stresses without leakage, sometimes even increasing the diameter of the vessels to get back to an average 15 dyne/cm^2^ shear stress [75]. In addition to aligning endothelial cells, flow and shear stress in the longitudinal axis also contribute to the cells’ apicobasal polarity in the radial direction of the tube, which enables the selective exchange of materials between the blood and the surrounding tissues [76].

Like the vascular system, the lymphatic system includes similar tubular vessels. Lymph is carried in vessels ranging from 10 μm capillaries to several millimeters-wide thoracic ducts [77]. While their dimensions are similar to blood vessels, they operate at speeds of only a couple of millimeters per second, leading to shear stresses 10 times lower [78].

Epithelial tissues constitute another large family of anisotropic tubular tissues, usually connecting the body to the outside world. For instance, milk is transported in the breast through 2 mm-wide lactiferous ducts [79], while nephrons filter and evacuate urine from the kidney in 60 μm channels [80]. In the digestive tract, food is progressively moved and transformed through 9 m of various hollow organs [81,82]. In addition to this motion and the apical-basal polarity of epithelial cells, another anisotropic feature of the gut is the radial orientation of crypt and villi structures, increasing the surface for nutrient exchange. Crypt and villi arise from mechanically driven buckling instabilities of the epithelium under compression from surrounding smooth muscle layers [83], illustrating how geometry and mechanics are tightly coupled in tubular anisotropy. After morphogenesis, the smooth muscles encasing the digestive tract apply a pressure between 70 and 200 mN/mm^2^, in peristaltic waves - traveling muscle contractions - that drive the intestinal bolus [84] (Fig. 3B). Smooth muscles also regulate directed movement in other hollow organs, such as the respiratory tract, the uterus, and throughout vasculature where they can apply 350 mN/mm^2^ pressure [85].

Mechanical forces are also responsible for bending and buckling of the neural groove into the neural tube, the hollow tubular structure from which the central nervous system arises during embryogenesis [86,87]. This tube persists in the shape of the spinal cord, spanning from the brainstem to the lower back. There, neurons run parallel around the central canal meant for nutrient and waste exchanges [88].

Altogether, tubular tissues impose combined requirements of lumen formation, radial-axial organization, and dynamic mechanical stimulation, which represent key design constraints for anisotropic tissue engineering strategies.

d. Porous tissues

While tubular tissues are characterized by a single oriented lumen, other tissues are characterized by a network of anisotropic pores, giving them a honeycomb-like structure, as well as unique mechanical properties.

The trabecular part of bone is filled with such pores, ranging from 10 μm to 1 mm [89] and oriented in the direction of the bone main load [90] (Fig. 3C and D). This makes it possible to drastically reduce the weight of bones, with a porosity above 75% [89,91], while preserving structural integrity thanks to an increased elasticity and stiffness [92], as well as resistance to cyclical fatigue [93] - material damage from repeated stress cycles - in the load bearing direction. Due to its spongy structure, shock absorption is trabecular bone's main mechanical function, which is also affected by the pores' anisotropy. Indeed, femur heads were shown to absorb more energy in the medial-lateral axis [94]. Permeability was also shown to be higher in the pores' main direction [95], which promotes anisotropic nutrient diffusion and likely facilitates vascularization, and overall cell migration in this direction [96]. These features indicate that engineering porous anisotropic tissues requires simultaneous control over pore orientation, size, and interconnectivity to couple mechanical support with transport properties.

Next to bone tissue, one of the most well-known porous tissues in the body is the lung, due to its hollow alveoli and branching structure. Although alveoli are often assumed to be isotropic, increasing evidence points out to their anisotropic morphology and behavior. Indeed, the average distance between airspaces in mouse lungs was shown to consistently be the highest when measured in the longitudinal axis of the lungs [97], and even more so when expanded. This also proved to be the preferential direction of gas diffusion in the alveoli [98]. Combined with alveoli anisotropic expansion [99], these elements highlight how the porous anisotropy of the lungs aims to favor the back-and-forth air movements through the respiratory tract.

e. Layered tissues

Some materials in the human body are composed of distinct layers, either alternating or different in composition, stacked together. This organization introduces directional differences between the stacking axis and each individual layer, resulting in layered anisotropy.

Cornea is a typical example of lamellar materials, having six sharply defined successive layers with completely different compositions and functions [100] (Fig. 3E). The epithelium is 50 μm-thick [101] and displays the lowest adhesion, to fulfil its barrier function, whereas the endothelium is a cell monolayer on the opposite end of the adhesion spectrum. The 500 μm stroma ensures structural integrity of the cornea [102,103]. This stacking results in an anisotropic compressive stiffness - resistance to deformation under a compressive load - four times higher than in plane stiffness [104].

In addition to fibrous anisotropy, the skin is also constituted of three main layers. The epidermis is filled with cells, mostly keratinocytes, and acts as a barrier [105,106]. The intermediate layer, the dermis, is a connective tissue a few millimeters deep [107], mostly composed of a collagen fibers network with a low cell density, whereas the deepest layer, the hypodermis, is characterized by adipocytes, along with vascular and nervous components.

Similarly, the cerebral cortex is composed of six layers. From the outer molecular layer, mostly composed of horizontal axons and dendrites, to the inner multiform layer, with a wide range of cell types and shapes, each region of the cortex has its own composition and function. For instance, layer IV receives sensory input while layer V sends motor output. Overall, this lamellar architecture of the cerebral cortex optimizes information processing [108].

Not all layered tissues display differences in layer composition, and some can simply be constituted of a repeating motif. Cortical bone is composed of a succession of compact concentric layers forming an outer protecting sheath, with an elastic modulus of about 20 GPa [109]. Thanks to this stacked structure, elasticity is also twice higher in the longitudinal axis than the transverse plane [110]. Moreover, to protect the bone from torsion, the orientation of the mineralized collagen fibers shifts from one lamella to the other [111].

Such a layer orientation shift can be observed in the outer region of the intervertebral disc, called the annulus fibrosus. It is composed of 15 to 25 concentric lamellae, each made of parallel collagen fibers resulting in anisotropic mechanical properties [112]. However, from one layer fiber direction changes by 60°, improving resistance to multiaxial stresses.

Instead of preventing complex stresses, smooth and cardiac muscles use orientation changes between cell layers to induce them. Smooth muscles form two orthogonal layers around tubular structures: inner cells follow the circumferential direction whereas outer cells align along the axis of the tube, to produce peristaltic waves [113]. In cardiac tissue, the orientation of cardiomyocytes gradually shifts from one layer to another, from +60° at the endocardium to −60° near the epicardium [114]. This twisting architecture ensures uniform stress distribution, that can efficiently eject 60 to 70% of the blood within the heart despite cardiomyocytes only contracting by about 15% [115]. Due to the gradual rotation of layers, cardiac tissue exemplifies a transitional case between layered and gradient anisotropy, which will now be explored.

f. Gradient tissues

Although gradient anisotropy shares similarities with layered architectures, instead of discreet layers, it characterizes structures where a specific parameter, such as cellular orientation or microfeatures geometry, changes continuously in one direction. In some cases, gradient anisotropy can combine with another type of anisotropy, as mentioned with the heart.

This also applies to skin, where in addition to layered organization, a gradient of the orientation of collagen fibers can be observed, especially in the dermis [107]. The fibers run parallel near the surface of the skin, but become progressively less aligned with depth [116]. This alignment likely enhances mechanical properties of superficial layers, more exposed to external stresses.

Gradual changes in collagen orientation can also be observed in articular cartilage (Fig. 3F and G). Fibers run parallel to the surface in the superficial zone and progressively rotate until becoming perpendicular in the deeper zone [72]. This also results in a gradient of elastic properties. The Young's modulus normal to the surface gradually increases 10 to 50 times from the superficial to the deeper zone, whereas the parallel modulus follows the inverse trend [43,44].

Other features can lead to a gradient of mechanical properties, such as pore size and density in cancellous bone, which progressively increase with distance from cortical bone [117,118].

Gradients can also be observed in tissues undergoing dynamic changes and remodeling, such as stiffness gradients in the matrix of scars and other fibrotic tissues [119]. In addition to stiffness variations, tumor environments also display a wide variety of gradients, such as oxygen or vascularization gradients [120,121], driving tumor invasion. Aside from pathological cases, the coupling of mechanical and chemical gradients is crucial during embryogenesis, as they guide spatially specific morphogenic cues [122].

These examples indicate that engineering gradient anisotropy requires precise spatial control over continuously varying properties, which remains more challenging than discrete patterning strategies.

Overall, there is a broad spectrum of anisotropic tissues in the human body. Distinct anisotropic characteristics often coexist within the same tissues. For example, bones are filled with anisotropic pores exhibiting a size gradient, while smooth muscles consist of two superimposed tubular layers. Anisotropic features arise either from cellular organization, as seen in cardiac muscle, or matrix properties, as in the skin. Moreover, they exist across multiple scales, from collagen fibrils to entire skeletal muscles, with microscopic features contributing to macroscopic properties. The anisotropic structures of these tissues are critical to their material and mechanical properties, which are closely tied to their functions. In particular, anisotropy often enables optimized load bearing, transport, or force generation along a preferred direction. Therefore, in order to faithfully reproduce these native tissues *in vitro*, and especially recapitulate their function, reproducing their anisotropic organization is essential. However, the diversity of anisotropic architectures - fibrous, tubular, porous, layered, and gradient - also implies distinct design constraints for engineering strategies. As will be presented in the next section, several approaches have been developed to engineer complex tissues and can be leveraged to create anisotropic structures. Starting with fundamental work on 2D cellular alignment, strategies with increased levels of complexity will be introduced, eventually leading us to the potential of magnetic based techniques for anisotropic tissue engineering.

## 3 Main tissue engineering techniques to replicate native anisotropy

Engineering anisotropic tissues relies on a wide range of strategies that differ not only in their technical implementation, but more fundamentally in the mechanisms through which anisotropy is generated. To provide a clearer and more consistent framework, these approaches can be broadly classified into four main categories based on their primary mode of action. First, material-driven strategies rely on the intrinsic properties or architecture of biomaterials, such as surface patterning or aligned fibrous scaffolds, to guide cellular organization. Second, assembly-based approaches generate anisotropy through the spatial organization of pre-formed biological building blocks, including cell sheets, spheroids, or microtissues. Third, fabrication-based techniques, such as moulding, microfluidics, or bioprinting, impose anisotropy during the construction process by controlling architecture, flow, or deposition pathways. Finally, force-mediated strategies exploit externally applied physical cues, such as mechanical, electrical, or fluidic stimuli, to induce directional organization within developing tissues. Together, these strategies provide the conceptual and technological foundation for anisotropic tissue engineering, while also motivating the development of alternative approaches, such as magnetic actuation, capable of addressing some of their limitations.

a. Material-driven strategies: from 2D substrates to 3D anisotropic meshes

Material-driven approaches represent some of the earliest and most widely used strategies to engineer anisotropic tissues, as they rely on the intrinsic properties or architecture of substrates and scaffolds to engineer constructs with anisotropic properties.

The first approaches for anisotropic tissue engineering started on 2D substrates and established some of the fundamental concepts for cell alignment. In these systems, anisotropy is primarily guided by material properties and surface features, providing local cues that cells interpret and follow. The impact of a substrate topography was first observed by R. Harrison in 1912 on chick embryo cells which displayed significant movement and alignment along the threads of a spider web [123]. This phenomenon was named contact guidance in 1945 b y P. Weiss, who observed a similar axon alignment on grooved mica [124] and later theorized that cells are able to sense and consequently react to the topography of the substrate [125].

**Fig. 4:**
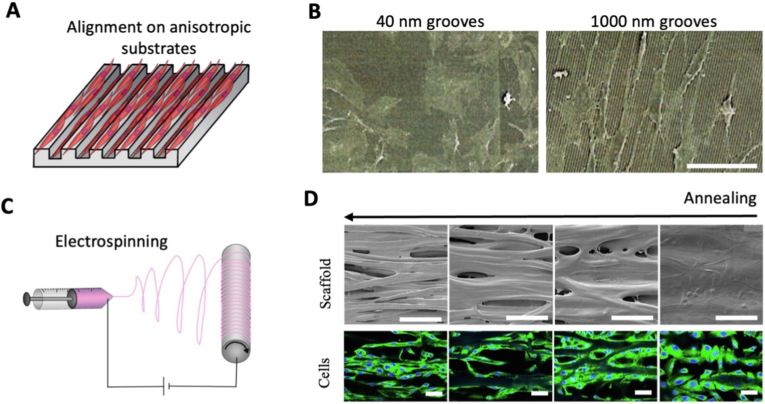
**Material-driven anisotropy engineering strategies.** (A) Illustration of cells aligning on an anisotropic substrate and (B) fibroblasts on substrates with 40 nm- or 1000 nm-wide grooves, imaged by SEM (scanning electron microscopy; Scale bar = 10 μm). Reproduced from Ref. [126]. (C) Illustration of electrospinning. (D) Electrospun scaffold with gradient vapor annealing and cell anisotropy (Scale bar = 50 μm). Reproduced from Ref. [127].

Advancements in microfabrication, notably photolithography and soft lithography in the 1990's, made it possible to further study this mechanism, starting by creating substrates with microgrooves (Fig. 4A). Early studies notably showed that while parallel microgrooves can promote cell alignment, this is conditioned by the geometry of the grooves [128]. Fibroblasts were shown to align only if ridges width was smaller than 4 μm [129]. Further technological advances enabled the engineering of nanogrooves no wider than 50 nm [126]. However, when the grooves were smaller than 80 nm, fibroblasts were no longer guided, showing that contact guidance works in a specific range (Fig. 4B). This highlights that material-driven anisotropy depends not only on the presence of directional cues but also on their scale relative to cellular sensing mechanisms. The response to microgrooves is also cell dependent [130]: unlike fibroblasts, Schwann cells alignment doubled when increasing microgrooves width from 10 to 20 μm or depth from 0.5 to 1.5 μm [131]. More complex geometries can even direct specific mechanisms, such as substrates with droplet shaped grooves which induced unidirectional migration of endothelial cells and favored vascularization when implanted in rats [132]. Microgrooves can even control the differentiation of myoblasts, as improved alignment has been shown to increase multiple myogenic markers [133,134].

Substrates’ anisotropy can also be induced by patterning adhesion molecules, using for instance by microcontact printing. In this case, a PDMS stamp with an anisotropic pattern is covered with matrix proteins, like fibronectin, and then pressed onto the substrate. Simply changing the surface chemistry to create an anisotropic pattern on the substrate, without impacting the microscopic geometry, still had a similar effect, and could induce alignment of human aortic vascular smooth muscle cells [135]. Here, anisotropy is driven by biochemical patterning rather than topography, illustrating that multiple material cues can independently guide directional organization. This technique can even be used with multiple coatings. For instance, this made it possible to create an aligned network of neurons where the soma was mainly on poly-lysine coating, whereas axons stayed on laminin patterns [136]. Moreover, the opposite effect of cell repulsion can also be achieved using anisotropic patterns of molecules with low protein binding like PEG (polyethylene glycol). Photolithography can also generate similar chemical patterning parallel lines in favor or against cell adhesion [137]. For instance, they were used to create cell sheets where more than 90% of myotubes were within a ± 10° orientation [138].

While 2D systems display a lot of diversity, even leveraging oriented meshes [139] or decellularized grass [140] to align cells, offering precise and reproducible control over cell alignment, they remain inherently limited by their planar nature. Extending these strategies to 3D constructs often requires stacking or transferring aligned layers, which can introduce additional complexity and limit scalability. As a result, although these approaches are highly effective for studying fundamental mechanisms of anisotropy and generating thin tissues, they are less suited for reproducing the full structural and functional complexity of native tissues.

To overcome these limitations, material-driven strategies have been extended to 3D fibrous scaffolds, most notably through spinning techniques. Electrospinning is an ideal method to create anisotropic scaffolds, and particularly fibrous anisotropy. An electric field between a polymer filled syringe and a collector causes the solution to jet out of the syringe, elongating into very thin fibers that dry and accumulate on the collector [141] (Fig. 4C). Electrospinning can now generate nanometric fibers similar to those found in native tissues out of biocompatible polymers, whether they are natural [142,143], synthetic [144], or semi-synthetic [145,146]. The geometry of the electrospun fibers can be controlled by parameters such as polymer properties, electrical field, or spinning distance. The most common way to align the fibers is to collect them on a rotating mandrel, with high speed inducing fiber alignment while low speed results in no alignment [127,145,147]. Fibers can also be aligned depending on the collector and electrode shapes [148], or the protrusions on the collector [149]. After cell seeding, the contact guidance cues induced by these parallel fibers, usually with a diameter of about 1 μm [127,147,150], will result in a similar cell alignment. Here, anisotropy is directly encoded within the scaffold architecture, providing a robust and scalable means to guide cell organization. Table 1 shows this technique has been particularly efficient to engineer tissues with fibrous alignment, whether the cells are tendon cells, cardiomyocytes [148], smooth muscle cells [147], or neurons [212].

**Table 1:** Table referencing various studies leveraging some of the most common techniques for 3D anisotropic tissues engineering, classified by the techniques employed and the tissue engineered.

	Spinning	Melt Electro-writing	Modular Assembly	Moulding	Microfluidics	Extrusion Bioprinting	Light-based Bioprinting	Stretching	Acoustic Forces	Electric Forces	Temperature Gradient
Skeletal Muscle	[151]		[31,138,152]	[[152], [153], [154], [155], [156], [157], [158], [159], [160], [161], [162], [163], [164], [165], [166], [167], [168], [169]]	[[153], [154], [155],^158^]	[24,25,[170], [171], [172], [173], [174]]	[175]	[31,157,[161], [162], [163],[175], [176], [177], [178]]	[179]		[170]
Cardiac Muscle	[148,149]		[148,149,[180], [181], [182], [183]]	[181,[183], [184], [185], [186], [187]]	[183,185]	[149,181,[188], [189], [190], [191]]	[192]				[185]
Smooth Muscle	[147,193,194]		[147,184,[195], [196], [197]]	[184,194,196]	[198]	[199]					[196]
Vascular	[127,145,146]		[183,197,200]	[155,183,185,[201], [202], [203], [204]]	[146,155,183,185,201,[203], [204], [205], [206]]	[171,189,206,207]	[192]	[208,209]	[210]	[211]	[185]
Nervous	[150,[212], [213], [214], [215], [216]]	[217,218]	[150,182,212,213,215,217,[219], [220], [221], [222]]	[219,220,223,224]	[158]					[219]	
Bone	[142,143,193]		[142,193]	[143,225]	[226,227]	[173,228,229]	[230]	[231]		[211,225]	[227,232]
Lung	[233]		[234]	[233,235,236]	[233,237]			[233,237]			[235]
Cartilage	[144]	[238]	[[239], [240], [241]]	[[242], [243], [244]]	[242,243]	[144,173,229,238,245,246]	[247]				
Skin			[248,249]	[250]	[205,250]	[249,251,252]				[253]	
Cornea			[[254], [255], [256]]	[254,255,257,258]		[[259], [260], [261], [262], [263]]	[264]				
Cancer			[221,222]	[204,265,266]	[204,267,268]						[269]
Kidney			[270,271]	[[272], [273], [274]]	[[271], [272], [273]]						
Tendon	[142,[275], [276], [277]]		[142]			[275,278]	[278]		[278]		
Annulus Fibrosus	[279]		[280,281]	[280,282]		[283]		[280,281]			[282]
Non-specific^a^			[31,[284], [285], [286], [287], [288]]	[286,289]	[284,289,290]			[31,290]	[291]		[290]

a Refers to studies applied to multiple cell types, or using non-specific cells or tissues, such as stem cells or NiH3T3 fibroblasts.

In addition to cell aligning properties, these electrospun scaffolds possess remarkable attributes. The strong coupling between fiber alignment and mechanical properties enables simultaneous structural and mechanical anisotropy, which is critical for load-bearing tissues. Similar to *in vivo* tissues, these aligned scaffolds display improved mechanical properties in the direction of alignment, with a longitudinal elastic modulus that can be ten times higher than in the transverse direction and three times higher than unaligned scaffolds [148]. This is crucial to ensure structural stability, especially when culturing muscle tissue. Shape memory properties [213] and gradients can also be induced within these fibrous scaffolds. For instance, solvent vapor annealing at only one end of the scaffold results in a gradient transition from anisotropy to isotropy, guiding endothelial cell migration and adhesion [127] (Fig. 4D). Both mechanical and chemical gradients can also be generated by progressively switching between two polymer solutions, which was used to modulate infiltration of cells from chick aortic arch explants seeded on electrospun scaffolds [145]. Scaffold properties can also be modified by adjusting spinning techniques. Instead of using a collector as an electrode, two oppositely charged syringes can simultaneously expel polymer filaments towards a central rod or funnel, enabling the twisting and braiding of multiple fibers into an anisotropic yarn filament [142,193].

Some spinning methods do not require electric forces. Filaments can be extruded and spun using centrifugal forces [214]. Microribbons could also be wet-spun resulting in microporous gels that facilitate cell encapsulation, which can be challenging for electrospun scaffolds [194]. For example, this approach proved to be particularly efficient to promote smooth muscle cells adhesion and alignment.

Another variant of electrospinning, which combines elements of 3D printing, is melt electro-writing (MEW). Instead of creating very thin fibers, a heated and charged syringe jets a polymer solution onto a collector oppositely charged [217,218,238]. While it is more time consuming and complex than electrospinning, this method can create very precise designs with fibers only 10 μm wide, and potentially toxic solvents are no longer needed. Although the fibers are larger, cells can still align along them to some extent [217]. They can also be used to create a frame, which will then be filled with a cell loaded gel [238], imbuing it with anisotropic mechanical properties, according to the elongation or spatial changes of the voxels [195], which has been particularly exploited for cartilage engineering.

Compared to 2D substrates, fibrous scaffolds provide a more physiologically relevant 3D environment and improved mechanical performance. However, they generally require post-seeding of cells and offer limited dynamic control once fabricated. In addition, while techniques such as MEW improve architectural precision, they remain relatively slow and technically demanding. Overall, material-driven strategies offer excellent control over microscale alignment and mechanical anisotropy but remain largely static and can be limited in terms of scalability and hierarchical complexity.

These limitations have motivated the development of complementary strategies that do not rely solely on material properties but instead generate anisotropy through the spatial organization of biological building blocks or through controlled fabrication processes, as discussed in the following sections.

b. Modular assembly approaches

**Fig. 5:**
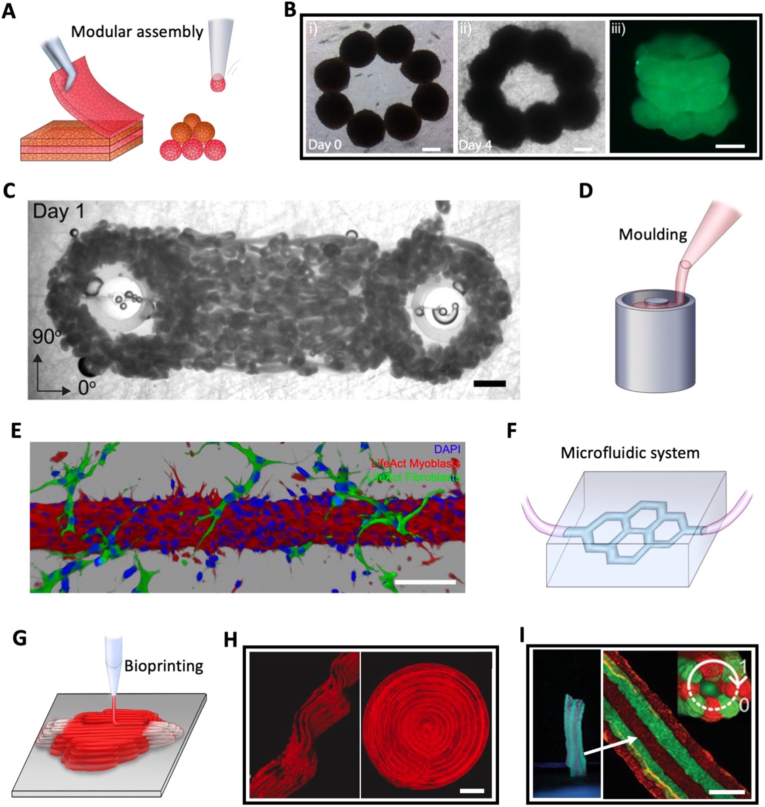
**Assembly- and fabrication-driven anisotropic tissue engineering strategies.** (A) Illustration of cell sheets and spheroids assembly and (B) MSCs spheroids pipette-positioned to form a tubular microtissue (Scale bar = 200 μm). Reproduced from Ref. [180]. (C) Dumbbell-shaped tissue bioprinted with a bioink loaded with smaller dumbbell-shaped cardiac microtissues (Scale bar = 1 mm). Reproduced from Ref. [181]. (D) Illustration of scaffold casting in a hollow cylindrical mould. (E) Muscle fascicle-like tissue, made with myoblasts seeded in a collagen channel moulded with a needle, with surrounding fibroblasts (Scale bar = 100 μm). Reproduced from Ref. [153]. (F) Illustration of a microfluidic system. (G) Illustration of extrusion bioprinting. (H) Bioprinted constructs with shear alignment of microfibers (Scale bar = 2 mm). Reproduced from Ref. [171]. (I) Bioprinted filaments with alternating bioinks (one labeled in green and the other in red; Scale bar = 2 mm). Reproduced from Ref. [170]. (For interpretation of the references to colour in this figure legend, the reader is referred to the Web version of this article.)

2D approaches have established the foundations for advancing 3D anisotropic tissue engineering, opening the door to more complex methods such as modular assembly, which has been applied to most types of anisotropic tissues, as shown in Table 1. These approaches enable the creation of more intricate and functional 3D tissue constructs by assembling biological building blocks (Fig. 5A), such as cell sheets.

This is particularly efficient to create systems with layers of different cell types, organization or composition. In this case, anisotropy is primarily introduced through geometrical stacking and controlled orientation of individual layers, resulting in structural anisotropy that can translate into directional mechanical and functional properties. To replicate the layered anisotropy of the cornea, stacking can be achieved using transwells, making it possible to have stromal cells at the bottom of the well while the epithelial cells are at the air-liquid interface [257]. A more direct approach consists of engineering cell sheets with the desired cell type on a robust, but detachable substrate, like polyethylene [254] or collagen [255] thin films, stacking them and then binding them together. Although more technically challenging, manipulating cell sheets has been facilitated by thermoresponsive materials, like poly (N-isopropylacrylamide) brushes which can enable cell sheets detachment when exposed to 20 °C for 30 min [138,248]. These surface treatments, in conjunction with microgrooves, can also be used to align cells, making it possible to stack layers with different orientation. Here, anisotropy originates from pre-aligned cellular organization within each sheet, which is then preserved and combined through hierarchical assembly to form tissues with layered or gradient anisotropy. This was used to reproduce the plywood orthogonal organization of lamellae in cornea, as well as in the annulus fibrosus [280,281]. Indeed, after implanting multilayered anulus fibrosus constructs in rat tails, the expression of collagen and aggrecan was twice as high in patterned tissues. Rotating layer orientation is also crucial for cardiac tissue engineering [149,248], where stacking cell sheets with progressively changing angles enables multiaxial contractions [148]. This represents functional anisotropy emerging from the coordinated orientation of contractile cells across layers. In smooth muscle, orthogonal stacking of cell sheets also enhanced contractile markers and calcium activity [195].

To reproduce peristaltic contractions in smooth muscle, layers not only have to be orthogonally stacked [147], but they also must be rolled into a tubular shape. In this case, anisotropy arises from a combination of layer orientation and geometrical transformation into a curved architecture, enabling directional mechanical function such as peristalsis. This can be achieved by transferring the cell sheets in a mould and fixing them with hydrogel [184]. If the cell sheet is soft enough, such as silk nanofibers scaffolds, a system similar to a rolling pin is enough to create the tubular shape. The direction of the rolling motion can change in between layers, and other cells can even be seeded in between layers, to innervate the junction between layers for instance [195]. Many approaches meant to recreate nervous tissue are also based on rolling cell sheets in order to create a tubular nerve guidance conduit, a structure reminiscent of the embryonic neural tube or the spinal cord. In addition to mechanical rolling [150,215], these approaches often rely on self-crimping scaffolds [212,213,217,218,292]. Because of the mechanical memory of materials like electrospun nanofibers, after being seeded, flat scaffold sheets can curl up and form a tubular shape when exposed to the right temperature or after a certain time. Here, anisotropy is partly encoded in the material itself and further amplified by the resulting 3D geometry. Layered and tubular anisotropy can also be combined by inserting a layered scaffold inside the tube [219], or by assembling multiple smaller tubes in a larger scaffold about to self-roll, resulting in a hierarchical structure similar to the one observed in a nerve with multiple fascicles [213]. These various nerve guidance conduit constructs, which are usually a few millimeters wide, not only displayed neural maturation *in vitro*, but when implanted in mice showed similar results to autografts, in terms of tissue colonization and functionality [150,212,213]. Nonetheless, these approaches depend on cell sheets that must be sufficiently robust to allow precise individual manipulation without breakage. As a result, scaffold materials are often selected primarily for their mechanical properties rather than physiological relevance, and there is a limit to the thinness of each layer, as well as the cell density within the scaffold.

Other pre-formed cellular constructs can be assembled together, like spheroids. They can be produced in many different ways, such as hanging droplet techniques, agarose wells or magnetic patterning [31,180,[292], [293], [294], [295], [296], [297], [298]]. Although isotropic individually, they can be assembled into larger, anisotropic tissues. In this case, anisotropy does not originate from the building blocks themselves but from their spatial organization and fusion after assembly. Complex geometries can be created by aspirating spheroids and then placing them with an automated micromanipulator in a support hydrogel (Fig. 5B), forming lines, rings, cylinders, pyramids, or any 3D shape [180,284]. Moreover, spheroids from different cell types can be assembled into the same structure, making it possible to study how they would fuse together or vascularize a structure in anisotropic configurations, for instance to model the asymmetry of cardiac fibrosis [180]. This enables the generation of compositional and functional anisotropy, for instance through directional vascularization or localized functional differences. While this approach offers high precision, it is quite time consuming and requires complex automation. Less precise but more scalable approaches include coordinated assembly methods such as magnetic assembly [31,239,240].

Similar to spheroids, microtissues can also be used as building blocks. Some are spherical [249], while others are already preformed into functional shapes [181]. They can then be incorporated into a bioink to be bioprinted into the desired macroscopic geometry. One study [181] even bioprinted 2 cm dumbbell-shaped tissues out of 2 mm dumbbell-shaped cardiomyocyte microtissues (Fig. 5C), enabling an initial optimization of the microtissues maturation before their assembly. Here, anisotropy is introduced both at the level of individual modules and through their spatial arrangement, resulting in multiscale structural organization. However, the complexity of the process, combined to added difficulty and lowered resolution, must be considered.

Textile-like weaving techniques also have the ability to achieve hierarchical anisotropy. Polymer nanofiber yarns are knitted or woven into repeating pattern. When seeded prior to assembly, cells can align along the nanofibers [193]. This leads to strong structural anisotropy at the microscale, which translates into directional mechanical properties at the macroscale. Moreover, different yarns can incorporate different cell types or compositions, enabling layered or gradient anisotropy. In this case, anisotropy is primarily dictated by the predefined architecture of the material rather than cell-driven alignment. For instance, weaving yarns with a gradient of hyaluronic acid led to gradual differentiation of bone marrow stem cells from tendon-like to bone-like tissue [142]. Similar to sheet-based assembly, weaving techniques depend on selecting an appropriate material composition and often involve complex weaving systems. However, these approaches rely on complex fabrication systems and often exhibit limited cellularization, making them more suitable for low cell-density tissues such as tendons.

Overall, modular assembly enables anisotropy through a combination of mechanisms, including pre-aligned building blocks, geometrical organization, and post-assembly cell remodeling. While this versatility allows the generation of structural, mechanical, and functional anisotropy across multiple scales, the technical complexity of assembly techniques can limit their throughput, reproducibility, and translational feasibility which is why many studies have aimed for simpler approaches, such as moulding, or automated platforms, such as bioprinting, which will be discussed in the following section.

c. Fabrication-driven techniques to engineer anisotropy

Fabrication-driven approaches generate anisotropy directly during the shaping of the construct. In these methods, anisotropy arises from imposed geometry, controlled deposition, or flow-induced organization, enabling precise control over spatial architecture and, in some cases, internal structure.

The most straightforward fabrication-driven strategies are likely moulding techniques, which can also engineer anisotropic materials for material- or assembly-driven approaches. However, moulding techniques are not limited to simply patterning scaffolds with microgrooves to align cells [159,184,196,220,225,254,255,257,258], but also extend to more complex geometries and macroscopic structures. Because of their versatility, moulding techniques have been applied to many tissue types, which can be observed in Table 1. Here, anisotropy is primarily dictated by external shape constraints and boundary conditions, making these approaches particularly effective for generating architectural anisotropy at different scales. Honeycomb patterns can be used to recreate lung alveoli [143,233] or crypts and villi moulds to mimic intestinal tissues [299]. To do so, a mould with the desired patterns, acting as a stamp, is needed. Such mould can be made using photolithography to create thick 3D pattern with micrometric precision [143,183,233], but the depth of the imprint is usually below 200 μm. One way to create deeper structures is to use micro-milled [299], or 3D printed [31,153] moulds. These can also be used for microscale 3D film thermoforming with very thin membranes, to recreate 3D spherical alveoli [235].

While these techniques create anisotropic geometries, they mostly focus on interfaces. Gels can also be moulded at the macroscopic scale to create fully 3D anisotropic tissues, such as cubes, cylinders, trileaflets or more complex shapes [193,219,244,250,282] (Fig. 5D). In most skeletal muscle tissue engineering approaches, cell-loaded gels are cast into centimeter scale moulds with a specific 3D anisotropic design [157,158,162,164,165,167,168]. In these systems, anisotropy is often indirectly generated through cell-mediated tension between anchors, highlighting the interplay between fabrication constraints and cell-driven organization. A heart valve could also be shaped via a multi-step injection moulding process of two gels with different cell types [187].

The internal structure of a moulded matrix can be regulated by the mould filling process, which can generate gradients in a hydrogel [201,242,243]. This introduces compositional and mechanical anisotropy, where spatial variations in material properties drive directional cellular responses. To do so, different pre-polymer solutions are prepared and fed into a mixing microfluidic system at varying speeds, so that the mould is first filled with one solution, which is then progressively replaced with the other. This was used to create a compression modulus gradient, ranging from 2 to 60 kPa, leading chondrocytes to produce more collagen II on one end, but more collagen X on the other [242].

The internal geometry of a tissue can also be controlled by moulding it from within. A simple method consists in inserting a needle in a gel prior to polymerization, resulting in the formation of a tubular channel within the matrix which is then connected to a microfluidic system to seed it. As seen in Table 1, this approach is well suited to cells with tubular or fibrous alignment, such as muscle [153,154], endothelial [155,204] or epithelial cells [272,273] (Fig. 5E). In this case, anisotropy emerges from geometrical confinement and subsequent cell alignment along predefined paths. In addition to fibrous or tubular anisotropy, concentric layers of different cell types can be achieved through sequential seeding [155]. Needles are easy to remove from a gel, but they can only create straight channels. Sacrificial objects embedded in a gel can be used to engineer more complex networks. For example, a vascular network could be made with a 3D printed carbohydrate template, dissolved after polymerization [185]. Similar results can be achieved by multiphoton ablation which can carve any shape within collagen gels [203,274], making it easier to generate more physiological architectures with enhanced resolution.

In addition to cell seeding, microfluidic flows can also be used to directly shape hydrogels and tissues (Table 1). Co-axial microfluidic systems can be used to create cell loaded tubular gel filaments. An outer endothelial cell loaded GelMA (gelatin methacryloyl) pre-polymer solution can flow around an inner Ca^2+^ ion solution, forming a lumen to recreate blood vessels [206]. Droplet based techniques could even induce radial stiffness gradients in such tubes to create radial phenotype shifts in ductal carcinoma models [268]. When transitioning from a narrow channel to a larger chamber, shear stress can also align collagen fibers during injection. Collagen chambers can even be formed with a progressive rotation of fiber orientation, which can then induce a corresponding change in the orientation of cells, such as endothelial or cancer cells [206,267]. Compared to moulding alone, microfluidics enables dynamic anisotropy through flow-induced alignment and mechanical stimulation, making it particularly relevant for reproducing functional anisotropy associated with fluid transport. Microfluidic systems (Fig. 5F) can not only reproduce the anisotropic geometry but also the anisotropic stresses in native tissues, such as shear stress or intraluminal pressure, even after tissue formation. Table 1 highlights this trend of using microfluidic flows to stimulate tubular tissues meant to transport various fluids. For instance, kidney cell channels moulded in collagen dilate when exposed to *in vivo* pressures [300], and similar systems reproduce vascular or lung mechanics [233,237].

Overall, microfluidic systems are very versatile tools for anisotropic 3D cell culture. Cells can be organized in separate compartments or connected through channels [158]. They can also be separated by thin membranes, which are useful for recreating lung structure [237], and can be exposed to a wide variety of stresses replicating *in vivo* conditions [233,237,300]. Cell-loaded gels are often used to fill chambers, moulded in various shapes, to recreate the native 3D environment [31], but they can also be used to create chemical or physical gradients between compartments, such as those present in skin [205,227]. These microfluidic chips meant to replicate native organ or tissue architecture were coined as “organs-on-chips” by D. Ingberg in 2010 [19,20] and are particularly useful for drug screening, disease modeling, and gaining biomechanical insight into a specific organ [16,19,301]. For example, a bone-on-chip with parallel and progressively smaller channels could demonstrate that smaller channels favor osteogenesis [226]. Leveraging other biofabrication techniques, such as electrospinning, could create aligned fiber networks within a vascular microfluidic chip, demonstrating that the impact of mechanical anisotropy outweighed chemical gradients in endothelial cell migration [146]. However, despite their high level of control and physiological relevance, these systems are typically limited to small-scale constructs and low cell numbers, restricting their direct applicability for large tissue fabrication.

While moulding and microfluidic approaches enable precise control over geometry and flow-induced organization, they remain constrained either by predefined templates or channel architectures. To further increase design flexibility and enable direct, programmable fabrication of anisotropic constructs, bioprinting techniques have emerged since the early 2000's as powerful approaches to create biological tissues with a wide range of possible geometries, making them applicable to most anisotropic tissues, as seen in Table 1. First pioneered by inkjet techniques [263,302,303], bioprinting has now shifted more towards extrusion platforms, and to some extent photopolymerization.

Extrusion bioprinting is akin to regular extrusion 3D printing, but replaces the plastic filament with a bioink, usually a cell loaded hydrogel solution, which is progressively extruded by pneumatic pressure onto a collector (Fig. 5G). During the process, the trajectory of the nozzle is controlled by a preprogrammed design, making it possible to precisely control not only the overall 3D shape of the tissue but also the positioning of each extruded filament within. In addition to printing parameters, the choice of bioink is essential, to balance adequate biocompatibility, viscosity and printability. Polymerization can occur during printing via UV curing, temperature changes, or ionic crosslinking [24,25,170,172,173,190,206,245]. If this exposure is not uniform, it leads to stiffness gradients within the printed tissue, which can be harnessed to mimic native gradients [252].

In stereolithography, light curing goes beyond fixing the shape of the tissue after printing, as it enables polymerization of bioinks layer by layer. In most cases, cells are seeded after printing as photoiniators can be cytotoxic [192,230]. Nonetheless, newer systems enable partial cell encapsulation with acceptable viability, notably using GelMA, for instance enabling crosslinking of a cell-loaded cornea scaffold with more than 75% viability [264].

In extrusion-based bioprinting, most approaches encapsulate cells beforehand, to improve density and spatial control, but as a result cells can be exposed to high shear stresses, affecting viability above certain thresholds. One study showed that H9C2 cardiomyocytes viability was impacted when shear stresses surpassed 80 Pa [190], while in another overall extrusion pressure had to be below 70 kPa to prevent the death of bone mesenchymal stem cells [229]. Interestingly, this constraint can also be leveraged to align polymer fibers and encapsulated objects during extrusion [171,181,190,207,260] (Fig. 5H). Shear induced alignment can be modulated with the viscosity of the bioink, the nozzle diameter [260] or aspect ratio [190]. Indeed, when extruding a bioink with approximately 20 mg/mL of collagen, at a flow rate of 0.0024 mm s^−1^ collagen fibrils would begin to show a clear aligned distribution when nozzle diameter was less than 0.5 mm.

Beyond controlling the anisotropy of the global shape and the alignment of the polymer fibers, extrusion paths can also induce anisotropy at the intermediate scale, by following parallel or perpendicular trajectories [171,172,189]. Alternating between bioinks can also generate anisotropy. Switching between a cellularized and a decellularized bioink can create parallel cellular rows within an acellular matrix, which can induce muscle cell alignment [25]. Rotation of these rows between layers can also reproduce the crisscross organization observed in the cornea [259]. The complexity can be further increased by using multiple bioinks that incorporate different cell types, enabling the formation of a bilayered osteochondral unit [229] or a intervertebral disk unit with distinct annulus fibrosus and nucleus pulposus regions [283]. In one study, scaffolds were made from alternating four different bioinks, one with a sturdier PCL (Polycaprolactone) scaffold, two with different cell types and one designed to degrade and create micropores [173]. Multi-nozzle systems further increase throughput and complexity, each nozzle dispensing at the same time a different gel [170] (Fig. 5I). Alternating layers can be achieved by switching bioinks within each filament, creating a striped filament [170], while radial layers are possible with a co-axial nozzle, to create a coculture core-shell structures [207] or tubular ones [206]. Nonetheless, with increasingly complex designs also come increasingly complex systems, that need to optimize the extrusion of different bioinks simultaneously, limiting throughput and speed.

In addition to purely material- and geometry-driven strategies, bioprinting is increasingly combined with auxiliary physical fields to further enhance anisotropy. For instance, electric fields, magnetic fields [174], or ice-templating approaches [170] have been used during or after printing to induce additional alignment of cells, fibers, or pores within printed constructs. These hybrid strategies, which will be discussed in the following sections, enable finer control over microstructural organization beyond the intrinsic resolution of printing alone.

Overall, fabrication-driven approaches provide strong control over geometry and spatial organization, with moulding excelling at macroscopic shaping, microfluidics enabling dynamic control of the microphysiological environment, and bioprinting offering high versatility. However, their ability to generate finely tuned 3D microscale anisotropy remains limited compared to material-driven techniques, and they often rely on additional cues to fully recapitulate native tissue organization.

This limitation has motivated the development of complementary strategies that directly impose anisotropic cues at the microscale through external forces, which are discussed in the following section.

d. Inducing anisotropic microfeatures with tissue scale forces

To form anisotropic microfeatures throughout a scaffold without having to generate them one by one, various forces can be applied at the scale of a whole tissue. Excluding magnetic forces, which will be discussed in the final section of this chapter, this includes mechanical stretching as well as remote forces such as acoustofluidics, electric forces and temperature gradients. In contrast to material- or fabrication-driven strategies, these approaches enable dynamic and often reversible control over anisotropy but may offer lower spatial resolution.

**Fig. 6:**
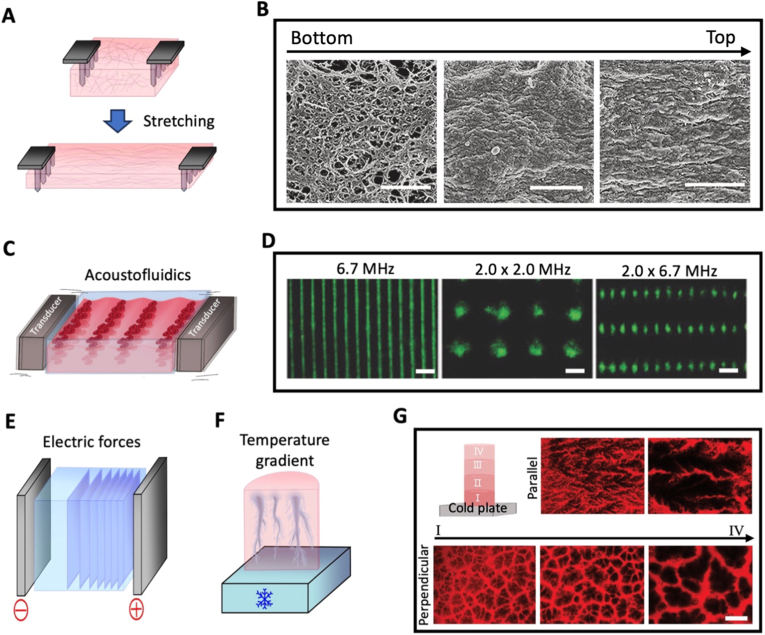
**Force-induced microfeatures**. (A) Illustration of scaffold mechanical stretching. (B) Incline-induced alignment gradient in chitosan gels, from bottom to top, imaged with field emission scanning electron microscopy (Scale bar = 1 μm). Reproduced from Ref. [209]. (C) Illustration of cell alignment in an acoustofluidic system. (D) Cellular patterns generated with acoustic forces using one or two pairs of piezo-electric transducers at varying frequencies (Scale bar = 200 μm). Reproduced from Ref. [179]. Illustrations of (E) nanofiber layers forming in a gel under an electrical field and (F) directed pores forming in a gel exposed to unidirectional freezing. (G) Longitudinal and transverse sections of a scaffold with gradient porous anisotropy formed by unidirectional freezing (Scale bar = 200 μm). Reproduced from Ref. [170].

Even when a scaffold is originally isotropic, mechanical forces, particularly stretching, can induce anisotropy (Table 1). Stretching deforms the matrix and aligns polymer fibers (Fig. 6A). This configuration can be preserved, especially if polymerization is incomplete during stretching, which can induce cell alignment, such as the formation of parallel blood vessels [208]. If a scaffold, still polymerizing, is stretched while inclined, it can even create a transition between aligned fibers at the top and disorganized ones at the bottom (Fig. 6B), leading to gradients in cell alignment and in stiffness from 22 MPa to 1 MPa in chitosan gels [209]. Similarly, porous anisotropy can also be achieved by stretching a scaffold during pore formation. Stretching a PCL scaffold during the extraction of its polyethylene oxide phase led to the formation of aligned pores with an aspect ratio of 4, leading to a 350% increase in collagen I expression after *in vivo* implantation to repair rat cranial defects [231].

Stretching can not only align scaffolds, but it also induces anisotropy by directly affecting cell behavior. Stretching drives cells to align, notably for skeletal muscle [31,157,[161], [162], [163],^176^], which likely explains the trend that most studies in Table 1 leveraging stretching aim to engineer muscle tissue. Although in static conditions cells tend to align with the matrix in the direction of stretching, under cyclical stretching other orientations, such as perpendicular or oblique ones can emerge depending on cell and loading conditions [304]. In fibroblast-loaded scaffolds, cyclical stretching can even provoke a gradual shift in cell orientation from perpendicular to the stretching on the tissue surface to parallel at the core [305,306]. However, control over final architecture remains indirect, as it depends on cell-matrix interactions and time-dependent remodeling.

With acoustofluidics, aligned cellular patterns can be created prior to polymerization. A cell loaded hydrogel solution is placed between piezo-electric transducers generating opposing ultrasound waves, driving cells towards the planes perpendicular to the pressure nodes [278] (Fig. 6C). This arrangement enhances anisotropic fusion of myoblasts into myotubes, with a tenfold increase in the MRF4 differentiation marker [179]. Additional pairs of transducers with different frequencies can be added to trap cells into more varied patterns, such as wavy lines or a network of rectangles (Fig. 6D). Although the range of achievable patterns with acoustofluidics is limited, moulds can be used to further guide the acoustic waves and therefore add another level of complexity to the possible structures, such as networks of adjacent vortexes [291] or 3D spirals [210].

Electric forces can create aligned layers of silk nanofibers within a scaffold [211,219,225,253], by forcing silk nanofibers, in a solution between two electrodes, to collect in planes perpendicular to the current direction near the positive electrode (Fig. 6E). After formation of the layers, freeze-drying is used to preserve this anisotropic configuration. Layer thickness, typically on the order of micrometers, increases with exposure time, enhancing compressive modulus in both directions but remaining higher along the layer orientation [211]. The geometry can also be adjusted by electrode configuration. Using cylindrical and rod-shaped electrodes forms concentric layers progressively more spaced along the radius [225], which can be used to replicate the transition zone between cortical and trabecular bone. Although cell migration and colonization can occur in a matter of days [219], these approaches are material-specific and often require post-seeding, limiting biological integration during fabrication.

Temperature gradients are widely used to generate anisotropic porous structures. Freeze-drying is a popular method to create micro-porous scaffolds from a wide selection of materials, such as collagen [269], silk fibroin [232] or chitosan [185] hydrogels. While uniform freezing results in an isotropic pore distribution, freezing from one direction induces a temperature gradient along one axis and pores elongate in this direction (Fig. 6F) This can then favor cell migration, making it possible to study tumor invasion for example [269]. Bioprinting onto a freezing plate enables the growth of lamellar ice crystals from the substrate, eventually resulting in honeycomb-like pores in the scaffold, even without lyophilization. This directional freezing not only orients the channels, but also results in a gradient of pore size, with pores getting larger the further they are from the freezing source [170] (Fig. 6G), which increases the elastic modulus in the pores’ direction and favors alignment of myotubes or endothelial cells. Additionally, pore size and distribution can be controlled by adjusting the overall temperature field and the scaffold composition. Temperature gradients can also induce thermophoresis, driving particles or fibers from hot to cold regions and generating stiffness gradients [227], which can guide osteogenic differentiation.

From mechanical to electrical forces and temperature gradients, a wide range of external forces can induce anisotropy at the tissue scale. These approaches are particularly powerful for rapid, large-scale patterning, but generally provide less precise and less stable control than material- or fabrication-based strategies.

Taken together, these approaches illustrate the diversity of strategies available to engineer anisotropic tissues, each relying on distinct mechanisms to guide directional organization. Material-driven techniques primarily leverage pre-defined structural cues to induce cell alignment through contact guidance, offering high reproducibility but limited adaptability once fabricated. Assembly-based approaches enable hierarchical organization through the spatial arrangement of pre-formed building blocks, providing greater architectural complexity at the cost of increased technical complexity and reduced scalability. Fabrication-based strategies, including moulding, microfluidics, and bioprinting, allow precise control over 3D geometry and spatial distribution of materials and cells, but often face trade-offs between resolution, throughput, and biological constraints such as cell viability or matrix compatibility. Moreover, approaches based on externally applied forces introduce dynamic and potentially reversible control over anisotropy, although they remain constrained by physical limitations related to force penetration and uniformity.

Rather than representing competing strategies, these techniques define a spectrum of design logics for anisotropy generation, ranging from static material encoding to dynamic external regulation. To clarify these relationships and facilitate cross-comparison between approaches, Table 2 summarizes the main characteristics, advantages, and limitations of each technique based on key criteria, including the mechanism of anisotropy generation, spatial control, scalability, and translational potential.

**Table 2:** Comparative table of the different techniques for engineering anisotropic living tissues. The reported spatial resolutions correspond to typical feature sizes achievable with each technique. Classifications of structural control, scalability, and limitations are based on representative implementations from the literature and are intended for comparative purposes.

Technique	Anisotropy Mechanism	Spatial resolution	Structural control	Multiscale integration	Scalability	Advantages	Limitations
**Electrospinning**	Material-driven (aligned fibers, contact guidance)	∼100 nm – 5 μm	Local → Intermediate	Moderate	High	High surface area, strong mechanical anisotropy, scalable	Limited control over global architecture, post-seeding required
**Melt electrowriting (MEW)**	Material-driven (precise fiber deposition)	∼5 – 20 μm	Intermediate → Global	Moderate–High	Low–Moderate	High precision fiber placement, controlled architectures	Slow fabrication, limited throughput
**Modular assembly**	Assembly-driven (geometric stacking, fusion, contractility)	∼50 – 500 μm	Intermediate → Global	High	Low	High biological relevance, hierarchical organization	Labor-intensive, low scalability, fragile modules
**Moulding**	Fabrication-driven (imposed geometry, confinement)	∼10 – 200 μm	Intermediate → Global	Low–Moderate	High	Simple, reproducible, compatible with many materials	Limited micro-scale control, mostly static structures
**Microfluidic systems**	Fabrication-driven (flow-induced alignment, gradients)	∼10 – 100 μm	Local → Intermediate	Moderate	Low	Precise control of gradients and dynamic environments	Limited size, low throughput
**Bioprinting**	Fabrication-driven (controlled filament deposition, shear alignment)	∼100–500 μm (extrusion)/∼10–100 μm (light-based)	Intermediate → Global	High	Moderate	Precise spatial control, multi-material capability	Limited resolution (extrusion), bioink constraints, shear stress
**Tissue-scale forces**	External field-driven (stress, acoustofluidics, electrical cues)	∼100 μm – mm	Local → Intermediate	Moderate	Moderate	Dynamic stimulation, mimics physiological forces, can produce tissue-scale microfeatures	Limited spatial precision, difficult to localize effects
**Cell self-organization**	Cell-driven (contractility, matrix remodeling, sorting)	Cell scale	Local → Global	High	High	High physiological relevance, adaptive, spontaneous	Limited control, variability, slower processes
**Magnetic alignment**	External magnetic field (particle/cell alignment along field lines)	Size of aligned objects	Local	Moderate	Moderate	Remote, rapid alignment, non-contact control	Presence of nanoparticles, limited depth uniformity
**Magnetic patterning**	External magnetic field (spatial positioning)	∼10 – 100 μm	Intermediate → Global	Moderate	Moderate	Precise spatial organization, programmable patterns	Field gradient limitations, complexity of setups
**Magnetic assembly**	External magnetic field (assembly of modules/cells)	∼50 – 500 μm	Intermediate → Global	High	Moderate	Rapid, remote assembly, versatility of assembly methods	Limited resolution, stability after assembly
**Magnetic actuation**	External magnetic field (stimulation, deformation over time)	∼100 μm – mm	Intermediate → Global & Dynamic	High	Moderate	Remote stimulation, reversible, tunable forces	Heating risks, field penetration limits, regulatory challenges

Importantly, this comparative framework also highlights that no single strategy fully recapitulates the multiscale and dynamic nature of native tissue anisotropy, which often emerges from the interplay between structural cues, cellular self-organization, and external stimuli. This observation motivates the exploration of complementary mechanisms, including cell-intrinsic reorganization processes (Section 4) and magnetic-based approaches (Section 5), which can further enhance or dynamically modulate anisotropic architectures.

## 4 Leveraging cells' self-reorganization for engineering anisotropic tissues

The methods described previously impose initial conditions on tissue architecture, either by precisely positioning cells or by strongly guiding and constraining them in an anisotropic manner. They actively rely on the cells' ability to sense and respond to topographical and mechanical cues. However, this response reflects only one aspect of the broader morphogenic capabilities of cells. Indeed, cells also have an intrinsic ability to self-assemble and self-organize [11,[307], [308], [309], [310]], independently of the initial scaffold. Gaining a deeper understanding of the morphogenic processes driving spontaneous cellular reorganization, even after the initial tissue structure has been established, is therefore essential for advancing anisotropic tissue engineering. In the following section, three key spontaneous cellular reorganization mechanisms, that can contribute to the formation of anisotropic structures, will be presented.

a. Spontaneous self-alignment

**Fig. 7:**
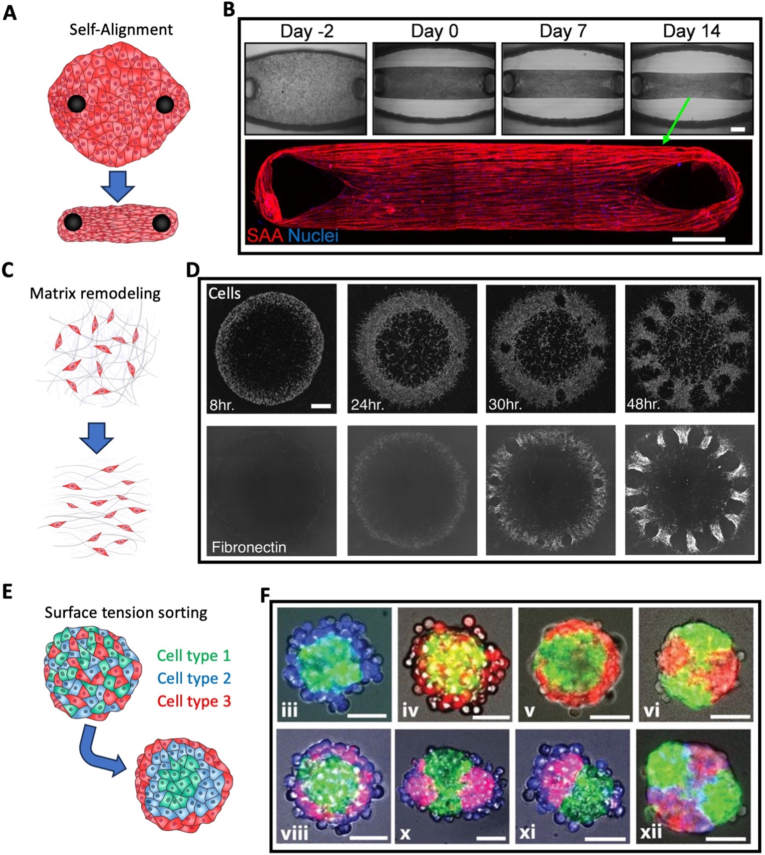
**Anisotropic tissue engineering approaches leveraging cellular spontaneous reorganization.***(A) Illustration of cellular self-alignment and (B) spontaneous alignment of muscle tissue constrained by two pillars, imaged over time with phase-contrast microscopy and at day 14 with confocal microscopy (Scale bars = *500μm*, alpha-actinin in red and nuclei in blue). Reproduced from* Ref. [166]. *(C) Illustration of matrix remodeling and (D) fibronectin scaffold remodeled by avian dermal fibroblasts into follicle patterns (Scale bar = *500μm*). Reproduced from Ref.* [311]*. (E) Illustration of surface tension cell sorting. (F) Various multicellular constructs made with two or three different cell types (labeled in blue, red or green), pre-programmed with the synNotch→adhesion toolkit to have different adhesion and tension surface properties to induce specific layering and cell sorting (Scale bar = *100μm*). Reproduced from Ref.* [312]. (For interpretation of the references to colour in this figure legend, the reader is referred to the Web version of this article.)

Cells can spontaneously align, both in 2D and 3D systems (Fig. 7A). It is particularly more apparent with skeletal muscle cells than any other cell type, as shown in Table 3, most likely because of their high contractility and aspect ratio.

**Table 3:** Table referencing various studies leveraging the cells spontaneous reorganization properties for 3D tissue engineering, classified by the type of reorganization and the tissue engineered.

	Self-Alignment	Matrix remodeling	Tension sorting
Skeletal Muscle	[156,160,161,166,168,177,178]		
Cardiac Muscle	[181]		
Vascular		[183,189]	
Nervous		[313]	[224]
Skin		[311]	
Cancer	[314]	[265,315]	[316]
Non-specific			[312,317,318]

On 2D substrates, differentiating myoblasts align to fuse into parallel myotubes. However, this self-alignment is only visible on areas of at most a few hundred micrometers, and at the macroscopic scale tissues appear isotropic. This self-alignment can be tuned by the shape and size of the boundaries of the substrate, as cells formed an aligned pattern on 400 μm-wide circles but a spiral on 1500 μm-wide circles [319]. Boundary constraints notably highlighted the tissue's correlation length, the maximum distance over which alignment propagates. Similarly, fibroblasts have the ability to form monolayers with aligned domains over a correlation length of about 500 μm. This organization was especially correlated to cell density, as aligned regions only appeared when cell density exceeded 50% [320]. Other factors, such as the cells' geometry and aspect ratio, can also impact cellular alignment [307,321].

In 3D, attaching a myoblast loaded scaffold to two pillars will automatically result in cells aligning between the two anchors [156,160,161,166,168], even if the gel is cast in an isotropic circular mould (Fig. 7B). Due to the cells’ contractility, longitudinal stresses develop between the anchors, whereas the absence of radial constraints only leads to deformation. This stress alignment leads cells to self-align, in an attempt to minimize deformation energy, a mechanism involved in several morphogenic processes [11,309,322], and very often harnessed in skeletal muscle tissue engineering.

Self-alignment in 3D scaffolds is not limited to myoblasts and has been observed in other cell types, including fibroblasts and cancer cells [314,319,323]. One common denominator of such self-alignment is cell contractility. This was evidenced in glioma cell-loaded gels suspended between two posts [314], where increasing contractility over time correlated with progressive alignment along the pillar axis. However, when integrin-mediated contraction was inhibited, cells no longer aligned and instead adopted an isotropic organization.

Overall, cell self-alignment refers to the ability of cells to align due to intrinsic properties, such as their contractility and geometry, in specific initial conditions, including boundary constraints and cell density. While this mechanism is a robust and often exploited source of anisotropy, it remains challenging to reproduce efficiently and typically requires external constraints to achieve macroscopic organization. Other processes, such as matrix remodeling, can further contribute to anisotropic tissue formation.

b. Matrix remodeling

Changes in the matrix often occur due to cells' ability to remodel it, a process frequently involved in generating tissue anisotropy (Fig. 7C). Although Table 3 shows that most studies leveraging this property usually involve cells with a high capacity for matrix remodeling, such as cancer or vascular cells, even cells that are not naturally contractile can remodel the scaffold's fibrils, causing the matrix to contract [313]. For instance, when a collagen scaffold, loaded with dental pulp stem cells and their neural derivatives, was constrained by two clamps, not only were the fibrils rearranged to align between the clamps, but collagen density also increased. Simulations have shown that such matrix remodeling processes are related to how cells interact with each other in a matrix. Fibrils density and alignment would particularly increase between two cells, to favor force transmission, cell migration and durotaxis [324]. However, this process can also lead to structural instabilities. For instance, when avian skin cells were patterned either in a straight or circular line, they would remodel the 3D matrix so that fibrils would follow the pattern [311]. This reinforced cell alignment and contractility, further enhancing remodeling and eventually leading to evenly spaced collapsed cell-matrix aggregates, reproducing avian skin follicle pattern formation (Fig. 7D).

While matrix reorganization is highly controlled in morphogenic processes, it is far more disordered in cancer tissues [325], making it an essential feature to reproduce for investigating the anisotropic tumor microenvironment. Tumors can first degrade the surrounding isotropic matrix with metalloproteinases. Then, contraction, extracellular matrix production and enzymatic crosslinking induce strain-hardening, which orients fibers radially, and generates anisotropic matrix compliance, lower in the extension direction of the tumor [315]. Moreover, such remodeling is closely associated with invasiveness. Expression of the Lox collagen-crosslinking enzyme is higher in the more aggressive MDA-MB-231 than the MCF7 cell line, resulting in a denser, more organized and anisotropic matrix architecture [265]. Anisotropic remodeling also promotes the migration of metastatic cells, as contractile cells further align fibers along their migration path [326].

Matrix degradation is also essential for angiogenesis [327], enabling endothelial cells to spontaneously sprout and gain a tubular organization. This tubular anisotropy can be guided *in vitro* by the geometry. For instance, in a microfluidic channel filled with an endothelial cell loaded gel, cells remodel the matrix to create a lumen, transforming a filled channel into a hollow vessel [183]. Similarly, in bioprinted lattices, endothelial cells tend to degrade the center of each filament, generating hollow channels whose orientation follows the printing path [189].

c. Surface tension sorting

In addition to self-alignment and matrix remodeling, anisotropic tissue engineering can benefit from spontaneous surface tension sorting of cells (Table 3). Cellular assemblies exhibit fluid-like properties, where cell-cell adhesion and contractility regulate an effective surface tension, driving self-organization to minimize deformation energy [11]. Differences in cell properties result in a wide range of surface tensions. Although both derived from the mesoderm, endothelial and mesenchymal stem cells form spheroids with very different surface tensions when measured with aspiration pipettes, 14 and 66 mN/m respectively [284].

In a tissue with a single cell type, surface tension will mainly regulate shape relaxation. If multiple cell types are mixed into a tissue, surface tension drives their respective organization, with cells usually forming consecutive layers from highest surface tension at the center to lowest at the periphery (Fig. 7E). For example, mixing cells from the ectoderm and the mesoderm of zebrafish would result in the ectoderm cells migrating towards the center and the mesoderm cells towards the edge [317]. However, when the mesoderm is replaced by endoderm tissue, having a higher surface tension than ectoderm, the situation is reversed [318]. Even with neurons and glial cells extracted from the same nervous tissue, neurons with a lower surface tension end up excluded to the edge of the spheroid [224].

Surface tension sorting can therefore be a very powerful tool to create a layered anisotropic architecture. Nonetheless, it remains mostly leveraged to study cellular reorganization in actively remodeling tissues, such as cancer or the primary germ layers, as shown in Table 3. Importantly, this mechanism is largely intrinsic and can occur unintentionally, potentially disrupting designed architectures if not accounted for. Control over cell sorting can be engineered by modifying cell adhesion or contractility. Inhibiting cell contractility can suppress sorting, while modulating myosin expression can even invert it [317]. Similarly, reducing *E*-cadherin expression decreases adhesion and leads to exclusion of these cells from the core, whereas increasing adhesion produces the opposite effect [316]. More advanced modifications of adhesion even made it possible to program cells to self-assemble into multi-layered structures that extend beyond spherical symmetry [312] (Fig. 7F).

## 5 Engineering anisotropic tissues with magnetic forces

Despite the remarkable advancements of current tissue engineering methods, whether they rely on active control of cell positioning and behavior or on spontaneous self-organization mechanisms, fully replicating the complexity of native tissues remains challenging. One approach that has displayed a lot of potential for anisotropic tissue engineering is the use of magnetic forces.

a. Magnetic nanoparticles in bioengineering

In magnetic tissue engineering, forces are mediated through magnetic micro- or nanoparticles, in particular superparamagnetic iron oxide nanoparticles. These particles have a core made of iron oxide, usually hematite α − Fe_2_O_3_, magnetite Fe_3_O_4_ or maghemite γ − Fe_2_O_3_, which can be as small as a few nanometers wide [328]. To ensure stability of the nanoparticles in aqueous conditions, the core is surrounded by a coating, the most commonly used being PEG, PVA (Poly (Vinyl Alcohol)), chitosan, PVP (Poly (Vinyl Pyrrolidine)), dextran or citrate [[329], [330], [331]]. The key feature of these nanoparticles is their superparamagnetism. Indeed, while bulk iron oxide is usually ferrimagnetic, but at the scale of the nanoparticles (usually of the order of 10 nm) it exhibits superparamagnetism: a magnetic field can induce magnetization, but no remanent magnetization remains once the field is removed, enabling precise and reversible control.

For biomedical applications, nanoparticles can either interact with the matrix or directly with the cells. In the former case, nanoparticles can be mixed into the matrix solution before polymerization [174,199] or spinning [214,275]. Nanoparticles can be coated to reduce leakage from scaffolds, to increase binding to the matrix or to cell membranes. For instance, streptavidin-coated particles can bind to biotinylated cells [332]. Nonetheless, the most common cell labeling is through nanoparticles internalization. Cells can viably internalize and confine superparamagnetic nanoparticles within endosomes [333], thereby acquiring magnetic responsiveness. Endocytosis varies based on cell type [334], as well as nanoparticles size and surface chemistry [335]. The level of iron internalization and consequently the magnetic moment of the cells also depend on nanoparticle concentration and incubation time [31,336], and can be measured through magnetophoresis [337].

Initially, magnetic nanoparticles were used as contrast agents for magnetic resonance imaging. In addition to their tendency to migrate towards inflammation and cancerous sites, they can be further functionalized to target specific tissues [338]. Moreover, tissues labeled with magnetic nanoparticles *in vitro* can be monitored through MRI after implantation, for instance making it possible to track labeled stem cells implanted into the mouse brain after more than a month [26]. These particles have also been explored for therapeutic applications, notably magnetic hyperthermia, where oscillating magnetic fields induce local heating for cancer treatments [[339], [340], [341]]. Since they were first used for tissue engineering purposes to magnetically form cell sheets in the early 2000's [27], magnetic based techniques have shown considerable progress, which will now be discussed in the context of anisotropic 3D tissue engineering.

b. Alignment along the magnetic field lines

One of the first ways to use magnetic forces to engineer anisotropic structures is magnetic alignment (Table 4). When superparamagnetic nanoparticles, or objects loaded with them, are exposed to a magnetic field, they gain a magnetic moment in the direction of the magnetic field lines and are subjected to a magnetic force proportional to their moment and the magnetic field gradient [31]. This results in their orientation and the migration along the magnetic field lines, already creating alignment [151,174,276,362,376].

**Table 4:** Table referencing various studies leveraging magnetic-based techniques for 3D anisotropic tissues engineering, classified by the techniques employed, the type of tissue engineered and whether the scaffold or the cells were magnetic.

	Magnetized scaffold				*Magnetically labeled* cells			
	**Alignment**	**Patterning**	**Assembly**	**Actuatio**n^a^	**Alignment**	**Patterning**	**Assembly**	**Actuation**^a^
Skeletal Muscle	[174,[342], [343], [344], [345]]			[175,346,347]	[31]	[31,169,177,178,348]	[31,152]	[177,178]
Cardiac Muscle				[191,349,350]	[329]	[29,351]		[352]
Smooth Muscle	[199]	[200,353]	[200,353]			[197,202]	[197]	
Vascular		[200,354]	[200]	[355]		[197,202,298,348,354,[356], [357], [358], [359], [360], [361]]	[197,298]	[356,360]
Nervous	[214,216,220,223,362]		[221,222]	[363]		[224]		[33,364]
Bone	[365,366]			[[367], [368], [369], [370]]		[359,371]		
Lung				[236]			[234]	
Cartilage		[244]	[239]	[244,370,[372], [373], [374]]	[375]	[240]	[240,241]	
Skin	[376]							
Cancer			[221,222]			[266,332]		
Kidney	[271]		[271]					
Tendon	[275,276]							
Non-specific	[377,378]	[286]	[[286], [287], [288]]	[289,378]	[31,379]	[31,285,380,381]	[31,285,381]	

a Refers to all methods meant to guide, stimulate or actuate a tissue after it has been formed.

This notably applies to magnetic micro-objects, such as nanoparticle loaded microgels or scaffold fibers. It is particularly efficient to align electrospun fibers, by placing a magnet near or below the collector [151,216]. Varying the distance to the magnet can even induce a gradient of fiber alignment, and consequently of cell alignment, which is ideal to mimic the structural gradients of musculoskeletal interfacial tissues [276]. Multiple magnets can be used to create even more complex gradients. Aligned chains of nanoparticles can also be generated during bioprinting. Indeed, the combination of the extrusion shear stress with magnetic forces can result in nanoparticles forming aligned chains in the direction of extrusion [199]. This could be achieved by passing the printing head through a ring magnet [174]. The more ring magnets used, the more anisotropic the scaffold becomes, which would directly impact the differentiation of muscle cells encapsulated in this bioink. Going from 1 to 3 ring magnets doubled the myotube fusion index and increased their maturation rate fivefold. These strategies leveraging magnetic alignment within usually non-magnetic approaches, such as electrospinning or bioprinting, showcase how magnetic forces can be used to complement them to further enhance anisotropic features.

When no flow or mechanical forces are applied to the gel, magnetic objects will migrate toward the magnets [31]. To prevent such aggregation, the magnetic field gradient must be null. This condition is met when the sample is positioned between two opposing magnetic poles [216,365], in a region where the magnetic field is uniform and the field lines run parallel. In this configuration, the field itself is present, but the gradient is zero, so superparamagnetic particles retain their induced magnetic moments and interact through dipolar coupling. At sufficiently high particle concentrations, these local magnetic interactions generate forces strong enough to align neighboring magnetic particles or objects into chains along the field lines [214,220,275,376]. When this alignment occurs within a prepolymer solution, subsequent polymerization locks the chains in place, resulting in a uniformly oriented microstructure throughout the scaffold. Such magnetically induced chain formation [220,362] has been demonstrated in systems containing electrospun PCL fibers, collagen bundles [342], or PEG microgels [343,376], leading to aligned architectures that promote cell organization through 3D contact guidance.

**Fig. 8:**
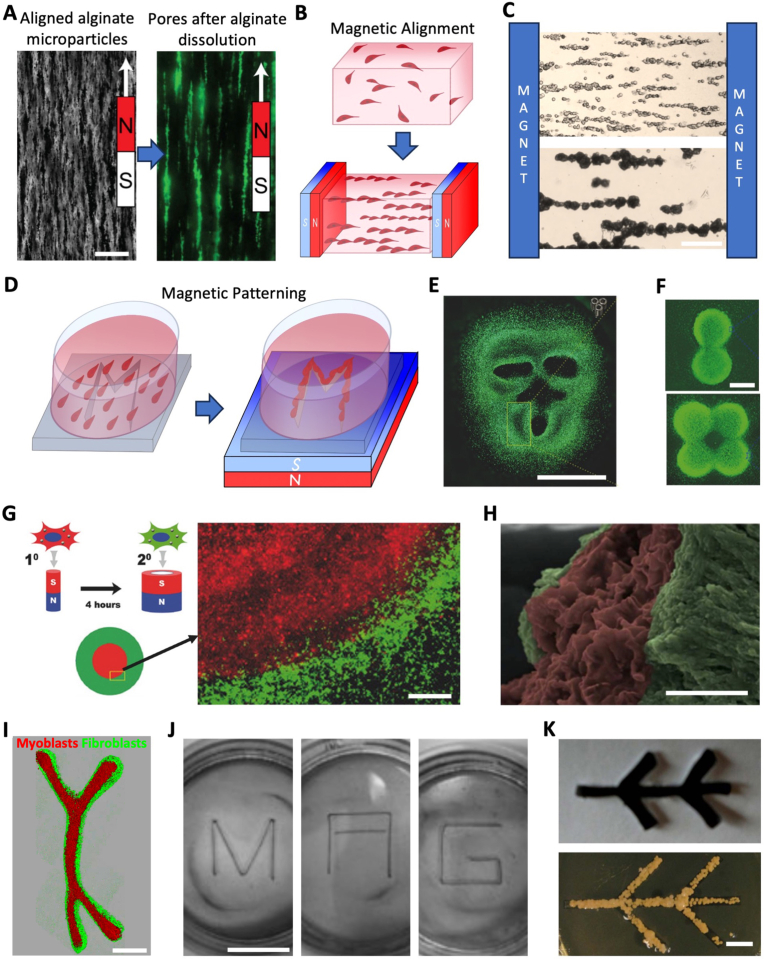
**Magnetic alignment and patterning.** (*A) Magnetically aligned magnetic alginate microparticles in gel, imaged with light microscopy (left), and the resulting aligned pores after alginate dissolution, imaged with confocal microscopy after soaking gel in dextran-FITC (right) (Scale bar = *500μm*, dextran-FITC in green). Reproduced from Ref.* [223]*. (B) Illustration of magnetic cell alignment. (C) Myoblast cells and spheroids, labeled with iron oxide nanoparticles, aligned under a magnetic field (Scale bar = *200μm*). Reproduced from Ref.* [31]*. (D) Illustration of magnetic cell patterning. Magnetically labeled cells patterned into (E) a face-like shape with three hollow cylindrical magnets (Scale bar = *5mm*) and (F) pixelized shapes with small cylindrical magnets (Scale bar = *2mm*). Reproduced from* Ref. [371]*. (G) Sequential magnetic patterning of two different cell types (labeled in red or green) using a small plain cylindrical magnet and a larger hollow cylindrical magnet to form a core-shell organization (Scale bar = *200μm*). Reproduced from* Ref. [371]*. (H) SEM imaging of HUVECs sandwiched between two layers of adipose-derived stromal cells by magnetic patterning (HUVECs in red, stromal cells in green, scale bar = *30μm*). Reproduced from Ref.* [359]*. (I) 3D visualization of a wrench-shaped tissue magnetically patterned with myoblasts with a strong magnetic labeling and fibroblasts with a weak magnetic labeling (Scale bar = *1mm*). Reproduced from Ref.* [177]*. (J) Letters “M,” “A,” “G” magnetically patterned out of fibroblasts (Scale bar = *1cm*). Reproduced from Ref.* [361]*. (K) Iron nanoparticle loaded spheroids, made of mouse yolk-sac endothelial cells, assembled with a magnetic pattern (Scale bar = *5mm*). Reproduced from Ref.* [298]. (For interpretation of the references to colour in this figure legend, the reader is referred to the Web version of this article.)

If the magnetic objects can be dissolved, this approach can be used to create anisotropic pores in the scaffolds. Magnetic alginate microparticles could be aligned in a hydrogel and then degraded with EDTA (Fig. 8A), resulting in a porous anisotropic scaffold with axons colonization comparable to that of isografts' after *in vivo* implantation [223]. Anisotropic pores can also be made by aligning magnetic nanoparticles when freezing an alginate gel, so that pores form under the guidance of the magnetic nanoparticles’ chains [382]. Although these methods do favor cell alignment, they both require seeding the gel from the outside after gelation.

While Table 4 displays less studies applying magnetic alignment on cells, some approaches have directly aligned them, without intermediary matrix components (Fig. 8B). Once cells have been labeled with magnetic nanoparticles, they behave like superparamagnetic dipoles when exposed to a magnetic field, and they can align in the same way as inert magnetic particles or objects [31,383]. For instance, magnetically labeled cardiomyocytes, myoblasts or even spheroids, can be mixed into a thermoresponsive Pluronic or collagen hydrogel solution at 4 °C, which is then placed between two strong magnets in an incubator at 37 °C. While the gel polymerizes, the cells or spheroids align and form chains, whose length is controlled by the magnetic labeling and the cell density [31,329] (Fig. 8C). More complex cell alignments can be achieved by polymerizing the scaffold step by step, while changing the orientation of the magnetic field. It can also be achieved directly with the proper magnetic field, such as a three-magnet system, which can also result in bent parallel field lines. This gradient or layered cell anisotropy applied to magnetic chondrocytes is ideal for reproducing the cartilage arch-like orientations [375]. Further configurations of cells alignment are possible with Halbach systems, such as a cylindrical chain of rotating magnets, which can create much more complex magnetic fields and gradients guiding the cells [379].

Overall, magnetic alignment techniques, whether they are applied to magnetically labeled cells or other microscopic objects, fall in the previously mentioned category of approaches relying on tissue-scale forces to induce the formation of anisotropic microfeatures, such as mechanical stretching [208] or acoustofluidics [179]. However, these platforms rely on direct physical contact with the tissue or polymer solution, affecting every element within them. Not only can magnetic forces generate microfeatures remotely, making it possible to align objects within systems or chambers otherwise difficult to reach, but they can also specifically target the magnetic elements without necessarily impacting the rest of the microenvironment [31]. If there is a need to upscale the size of the tissue, while the remote nature of these forces enables their application over large objects, generating uniform magnetic fields over extended areas will nonetheless require larger and more complex magnetic set-ups.

c. Shaping tissues through magnetic patterning: a bioprinting-like approach

While dipolar interactions are ideal to create arrays of aligned microscopic structures, classical magnetophoretic forces can also be used to pattern cells or microtissues into anisotropic structures (Fig. 8D). Unlike magnetic alignment, this technique is most often used directly on magnetically labeled cells instead of elements of the scaffold, as shown in Table 4.

When seeding magnetically labeled cells onto a scaffold in the presence of a magnet, this makes it possible to attract them to a specific region, creating an asymmetric or heterogeneous distribution of the cells [354,356]. If the magnet is placed below the scaffold, magnetically labeled cells in a suspension or in a pre-polymer solution will naturally migrate and aggregate in the shape of the magnet [380]. Thus, anisotropic magnets yield anisotropic constructs [286]. One study used star-shaped magnets to create a star-shaped cellular pattern [332], but geometries are usually limited since most magnets are parallelepipedal, cylindrical or ring shaped. Magnetic ring patterning is still particularly interesting to reproduce tissues with radial anisotropy like smooth or cardiac muscles [29,353]. For instance, magnetically patterned ring-shaped cardiac tissues displayed sarcomeric organization, coordinated beating, and could be implanted and monitored by MRI. Despite the 2D nature of the magnet surface, aggregates are inherently 3D due to cell stacking [31,357].

Pattern complexity can be increased by using multiple magnets. For instance, three ring magnets were used to aggregate cells into a face-like pattern (Fig. 8E). Arranging small magnets enables a “pixelated” approach to build defined geometries [371] (Fig. 8F). In specific cases, diamagnetic cells such as red blood cells can also be patterned in paramagnetic media into anisotropic shapes like lines or stars using multiple magnets [358].

Sequential patterning can further expand design flexibility. A first cell type can be patterned, followed by another using a different magnet geometry, enabling layered or core–shell structures [371] (Fig. 8G). Even without changing the magnet, sequential seeding can result in various layered anisotropies, with the possibility of asymmetrical core-shell structures depending on the direction the different cell types are brought next to the magnet [381]. When cells are consecutively seeded on top of the magnets, magnetic patterning can also create multilayered constructs. This made it possible to sandwich a layer of HUVECs between two layers of adipose derived stromal cells (Fig. 8H), enhancing osteogenesis [359]. A similar sequential seeding can be applied in a hollow cylindrical magnet, making it possible to engineer a multilayered anisotropic tubular tissue [202]. To create tissues with multiple cell types separated in different regions or layers, magnetic segregation can also be leveraged: differences in magnetic labeling can drive spatial separation of cell types in a single step, controlling both global shape and internal organization [177] (Fig. 8I).

Magnetic forces can be combined with other patterning or moulding techniques. These approaches provide geometrical guidance, while magnets can force cell aggregation [224]. For example, combining a central pillar mould with a magnet yields dense ring-shaped tissues that further compact during maturation [169,351]. However, magnetic moulding is not limited to the ring shape. Moulds fabricated by photolithography can guide magnetic aggregation with a 150 μm resolution, after which a second cell type can even fill remaining regions [266]. Surface patterning using magnetic PEG can also control adhesion and enables sequential co-culture organization [384]. Although a broader range of architectures can be engineered with these combined approaches, they raise significantly the level of technical complexity.

To achieve intricate geometries using only magnetic forces, the magnetic field gradient needs to be precisely controlled. Some have used neodymium magnetic sheets cut-out in the desired shape [298], while others steered towards magnetizable patterns that showcase a higher resolution. Placing a vertical thin steel plate between a magnet and a solution of magnetically labeled cells drives cells to aggregate along the line of the edge [240], making it possible to engineer very thin vascular channels on top of homogeneously distributed non-magnetic muscles cells [348]. Although the steel plate is not magnetic on its own, when exposed to a magnetic field, it concentrates both the magnetic field and gradient, especially near edges, resulting in magnetic forces much higher than those near a magnet [31]. Creating magnetic patterns out of the edges of these steel plates can therefore enable many other types of magnetic anisotropic aggregations, such as the formations of letters out of cellular aggregates [348,361] (Fig. 8J). Magnetic templates can also be made with photolithography and NiFe electrodeposition, enabling the formation of magnetic patterns with a micrometric resolution for cell aggregation [31,385].

Compared to other patterning techniques, magnetic strategies dispense with the need to precisely controlling the geometry or surrounding environment of the cells or tissues, as is the case with 2D grooved [129] or chemically patterned substrates [136], and 3D moulding techniques [153]. While the level of complexity of achieved structures is not yet on par with extrusion bioprinting [386], magnetic patterning holds the unique advantage to create complex structures solely out of cells, without the need for matrix. This could enable a better understanding of cell-cell interactions in 3D tissues, as well as highlight, through its absence, the role played by extracellular matrix. Moreover, matrix could be added later, with the possibility to precisely control when and how to add it to the magnetically patterned tissues.

d. Assembling cellular building blocks with magnetic forces

Magnetic patterning is not limited to individual cells and can also assemble multiple magnetic microtissues or spheroids together (Fig. 8K–Table 4). They can first be made using methods such as agarose wells [298] or magnetic aggregation [31,240], and then placed onto a magnetic pattern [197,240,285,286,298]. After assembly, cellular remodeling usually leads to fusion into cohesive tissues. The size of spheroids or microtissues is adjustable, enabling control over the tissues’ internal microfeatures and their 3D aspects. Magnetic labeling can also be performed during spheroid or microtissue formation [200,239]. Indeed, instead of labeling cells, magnetic nanoparticles can be mixed with matrix material, which becomes trapped between cells during their aggregation. However, since these objects are larger than cells, drag forces are higher, which can hinder the patterning process unless the system is regularly shaken [298]. Similar considerations are also at play when magnetically aligning modules within a hydrogel [31], with asymmetries in module shape or particle distribution sometimes leading to wave-like organizations [271].

**Fig. 9:**
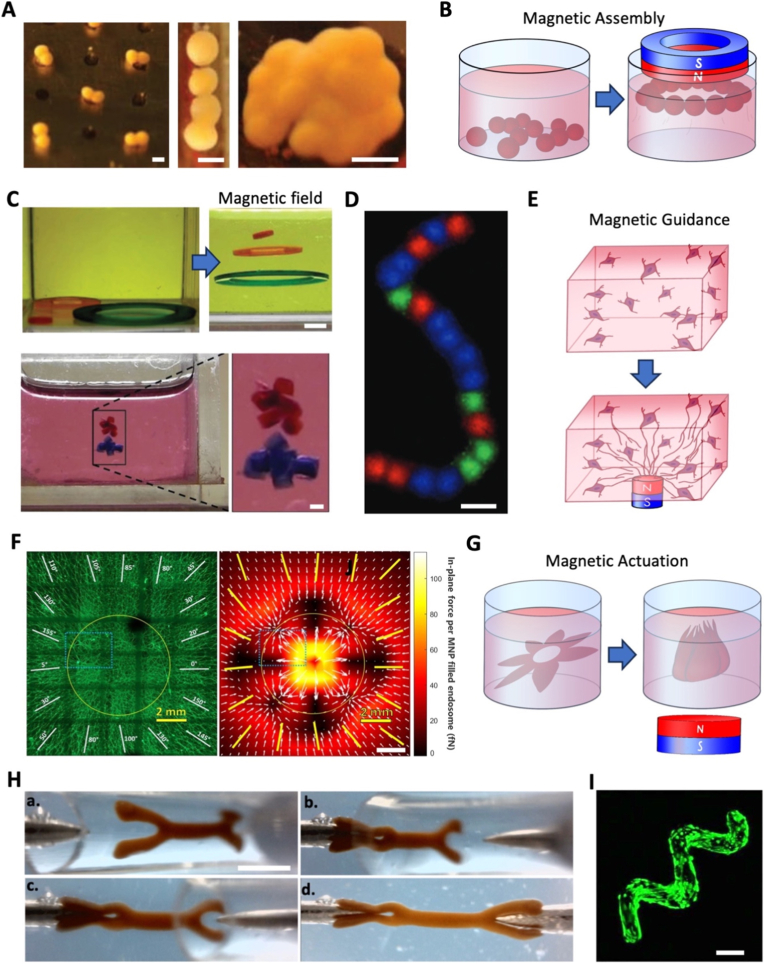
**Magnetic assembly, guidance and actuation.***(A) Cellular constructs engineered by magnetically induced fusion of MSCs spheroids (Scale bar = *1mm*). Reproduced from Ref.* [240]*. (B) Illustration of spheroid assembly by magnetic levitation. (C) Positioning and assembly of disk-shaped (top) and brick-like (bottom) hydrogels* via *magnetic levitation in paramagnetic medium, regulated by the differences in density of the components (Scale bar = *1mm*). Reproduced from Ref.* [287]*. (D) Assembly of a chain of spheroids made from three different cell types (labeled in red, green and blue) by magnetic levitation (Scale bar = *100μm*). Reproduced from Ref.* [288]*. (E) Illustration of magnetic cell guidance. (F) Map of the neurite network orientation of magnetically labeled SH-SY5Y cells, compared to the map of the magnetic force field applied during culture (Scale bar = *2mm*). Reproduced from Ref.* [33]*. (G) Illustration of a tissue folding by magnetic actuation. (H) Magnetic trapping of a cellular tissue magnetically patterned into the shape of a wrench with two clamps so that it can clip onto two magnetic needles (Scale bar = *1mm*; letters represent the chronological order of the sub-panels). Reproduced from Ref.* [178]*. (I) Fibroblast loaded magnetic hydrogel micromotor (Scale bar = *80μm*). Reproduced from* Ref. [289]. (For interpretation of the references to colour in this figure legend, the reader is referred to the Web version of this article.)

Magnetic alignment and patterning aside, the assembly process can be achieved by magnetic manipulation. A magnet can be used to bring spheroids together, sometimes with additional magnets to stabilize them during fusion [240] (Fig. 9A). This notably enabled the fusion of magnetic mesenchymal stem cell spheroids into cellular rods, which significantly increased cartilage functionalization [240]. In the case of magnetic sheets, a cylindrical magnet can also be used to easily roll the tissue into a tubular construct [152]. More advanced systems allow automated magnetic picking and placement of spheroids [221,292], enabling the construction of complex architectures such as rings or pyramids out of alternating ventral or dorsal forebrain neural organoids [221]. Tumor directed progression could even be modeled by assembling organoids from three different brain regions from the same patient, spanning from the origination site to metastasized tissue. Sequential picking without release can further generate anisotropic layered constructs [286,292].

Assembly can also be achieved through magnetic levitation (Fig. 9B). As individual cells can be magnetically levitated to form spheroids, spheroids can be levitated to assemble larger tissues [197,222,234,239,287]. If different objects are successively levitated, it will result in multilayered tissues. Such magnetic levitation not only made it possible to assemble four layers of epithelial, smooth muscle, pulmonary fibroblasts and pulmonary endothelial cells, but it could also maintain the resulting tissue at the air-liquid interface [234]. While conceptually similar to patterning, magnetic levitation is achieved by placing magnets above the cell suspension. It reduces the impact of mechanical compression but requires stronger magnetic forces to counteract gravity. However, the most complex structures typically achieved are often ring-shaped [222]. The density of the matrix of microtissues or hydrogels can be used to further control their levitation and therefore assembly [287] (Fig. 9C). Some studies have tried to use more intricate 3D systems of magnets to precisely control microtissues 3D assembly [241], even attempting it in space to minimize the bias of gravity [239]. While the range of possible geometries remains limited, microtissues were still able to viably fuse together into anisotropic 3D cartilage tissues in these more advanced levitational systems. An approach to achieve other forms of magnetic levitation is to use a paramagnetic medium [287,288]. Increased control can also be obtained by restraining the environment of the levitating objects, making it possible to trap a queue of levitating spheroids made from different cells in a channel to induce their anisotropic fusion [288] (Fig. 9D). Although 3D magnetic levitation assembly remains challenging, it could lead to a truly 3D form of magnetic bioprinting where biological building blocks are levitated and positioned to form precisely defined geometries.

Setting aside the remote nature of the forces involved in magnetic assembly, this set of techniques could reduce the complexity of the manipulation usually necessary when assembling non-magnetic cellular building blocks. Magnetic forces can provide a more gentle and controlled handling of the modules that otherwise have to be aspirated [180], woven [193] or extruded [181], with the possibility to turn down or even off the magnetic field if necessary. Moreover, the idea of collectively assembling tissues in a single step with the right magnetic field, as illustrated by magnetic levitation approaches [197,222,234,239,287], could greatly diminish the complexity of the experimental process. Experiments in space [239] even showed that it was possible to automate this process to some extent in less favorable experimental conditions, by simply letting spheroids assemble in the desired magnetic field with minimal outside manipulation. However, achieving assemblies of numerous objects in complex and precise configurations remains challenging and will require systems capable of much finer tuning of the variations of the 3D magnetic gradient, potentially including dynamical control.

e. Stimulating and moving tissues with magnetic actuation

In the various approaches so far, magnetic cells and tissues have been presented primarily as physical objects manipulated by magnetic forces. In contrast, magnetic actuation, also referred to as magnetic drive, designates the use of magnetic fields to actively apply forces or deformations to cells and tissues over time, thereby introducing a dynamic dimension to anisotropic tissue engineering. Indeed, even after scaffold polymerization, magnetic forces can influence cell migration and behavior (Fig. 9E–Table 4).

Within a polymerized matrix, neurons labeled with magnetic nanoparticles tend to extend neurites and migrate towards magnets, leading to the formation of anisotropic neural tissue [364,387]. Schwann cells and astrocytes, that usually do not mix with one another, can even be made to intermingle under such magnetic cues [364]. Magnetic guidance was proven to be effective over areas larger than 1 cm^2^, even with forces as low as 10 fN per magnetic nanoparticle-loaded endosome. Using multiple magnets enabled the control of neural networks, as neuroclusters would localize where magnetic forces converge [33] (Fig. 9F). Similar increases in migration and proliferation towards magnets can be observed with stem cells or endothelial cells, promoting the radial vascularization of tubular constructs [356,360]. While magnetic forces are applied on cells, they do not directly displace cells firmly attached to a substrate or matrix. However, magnetic forces act on magnetic endosomes within the cells, triggering mechanotransduction pathways and favoring cell motility in a specific direction [383].

Similar mechanotransduction cues can also be achieved through the actuation of magnetic matrices. Table 4 shows that this has been particularly applied to stimulate engineered tissues undergoing mechanical stress *in vivo*, such as muscles, cartilage or lungs. Indeed, magnetic forces can pull on a magnetic scaffold and consequently stretch it, which can favor muscle cell alignment in the stretching direction, as well as promote their differentiation [175,347]. On the other hand, tissues meant to resist anisotropic compression, such as bones, show improved differentiation under magnetic compression, achieved by placing a magnet under a magnetic scaffold [[367], [368], [369]]. Magnetic stimulation of the matrix alters focal adhesion, activating integrin signaling pathways in osteoblasts, resulting in improved osteogenesis markers such as increased alkaline phosphatase activity and mineralization. Cyclic magnetic pressure can be applied to tissues that endure dynamic loads *in vivo*, such as cartilage scaffolds displaying better chondrogenesis under an oscillatory magnetic field than a static one [372,373]. This highlights one of the key advantages of magnetic drive compared to most scaffold-based approaches, namely the ability to apply time-dependent and reversible mechanical cues that more closely mimic physiological loading conditions. Magnetic actuation can also deform membranes, forcing a magnetic hydrogel to alternate between a flat and a curved configuration, reproducing cartilage *in vivo* stresses [373,374]. Pre-conditioning can even result in membranes that fold into wave like patterns to mimic the regular dilation of epithelial lung tissues [236].

In addition to temporal variations, spatial heterogeneity could further reinforce the anisotropy of the tissue. For instance, applying a magnetic field to a scaffold bioprinted with alternating magnetic and non-magnetic bioinks results in a layered anisotropy of the stress field [388]. Stiffness gradients can also be engineered in magnetorheological elastomers, which can stiffen under magnetic forces, simply due to the progressive decrease of the intensity of the magnetic field with the distance from the magnet. This made it possible to model the dispersion of the fibrotic reaction of cardiac fibroblasts during an infarct, localized at the site of the magnet, and to observe the spatiotemporal entanglement of the fibrotic response [349]. The orientation of magnetic objects within the matrix also impacts stiffening. When exposing a matrix to a magnetic field, if the magnetic rods within are pre-aligned with the field lines, the matrix can become up to three times stiffer than with unaligned rods [377]. Aligned magnetic objects in a scaffold will also heat more if they are parallel to the alternating magnetic field, making it possible to create an anisotropic response to magnetothermal stimulation [389]. However, these effects highlight potential limitations, as local heating must be carefully controlled to avoid adverse effects on cell viability.

The impact of the alignment of magnetic microfeatures can also be used for directed movement of shape morphing hydrogels (Fig. 9G–Table 4). For instance, a cell loaded matrix shaped like a hand will bend towards a magnet if the magnetic nanoparticles were previously aligned, whereas it will not be affected if the particles are isotropic [378]. This enables the development of biohybrid actuators. With the right geometry they can bend to stroke [390], morph into a more compact shape [346,391], or even assemble on demand [191]. By adding two clamps to the geometry of magnetically patterned cellular tissues, one study showed that they could also clip themselves onto two magnetic needles in an automated fashion (Fig. 9H) [178]. These soft living robots are often designed to be remotely guided towards a specific location, with movement within the human body often in mind. Once a hydrogel robot has reached its goal, the cells it contains can proliferate, mature and intermingle with native tissues for biomedical purposes, such as a magnetic helical micromotor loaded with cells, capable of moving through tubes for vascular repair [289] (Fig. 9I).

As has already been highlighted, the key feature of magnetic approaches is the possibility for remote actuation. Unlike most conventional approaches that primarily generate static anisotropy, magnetic drive enables dynamic, spatially resolved, and reversible control over tissue organization and mechanical stimulation. In most conventional systems, post-fabrication stimulation remains limited and non-specific. Magnetic forces can remotely stimulate magnetically labeled cells or components, even at the core of the tissue, making it possible to guide their movements in complex fashions [33] and apply varied mechanical cues throughout culture time [349], even after implantation. Moreover, with the emergence of 4D bioprinting, control over tissue movement and actuation over time is essential and has mostly been achieved so far only via magnetic-based strategies [346,391]. However, precise calibration of forces and long-term biocompatibility remain important challenges for translation.

f. Translational challenges of magnetic approaches for anisotropic tissue engineering

A key challenge for the clinical translation of magnetic-force–based anisotropic tissue engineering lies in the use of magnetic materials, most commonly iron oxide, either in close contact with or internalized by cells. While magnetic nanoparticles enable complex remote and dynamic manipulation, their presence raises biosafety considerations that must be carefully addressed. Indeed, studies have shown cytotoxic effects of superparamagnetic iron oxide nanoparticles due to their surface charge [335]. Nanoparticles associated ferroptosis was also observed in cardiac cells [336], and even leveraged for cancer treatments [340]. These adverse effects can be due to the presence of positive charges on the nanoparticles, which can be addressed with the appropriate coating. For instance, citrate coating of nanoparticles leads to surface charges below −30 mV [31,178], promoting stabilization and minimizing non-specific interactions with cells, therefore favoring biocompatibility. In a previous study, citrate coated magnetite nanoparticles notably did not impact *in vitro* cell cultures, and even when injected *in vivo* no toxicity, nor organ morphological changes were observed, aside from iron accumulation in the liver [333].

Another potential cause of adverse effects is the release of ferrous iron (Fe^2+^), which can trigger the production of cytotoxic reactive oxygen species (ROS) through Fenton reactions. This effect requires nanoparticles that contain Fe^2+^ in their structure, which is the case for magnetite (Fe_3_O_4_), but not for maghemite (γ-Fe_2_O_3_). Even in the case of magnetite, surface oxidation quickly leads to the formation of a maghemite shell, preventing further oxidation. *In vitro* studies have shown that appropriately coated SPIONs can be internalized by cells while maintaining viability, proliferation, and differentiation capacity [392]. Moreover, intracellular nanoparticles can gradually degrade through physiological iron-handling pathways involving ferritin and lysosomal processing, suggesting that long-term persistence may be limited [393,394] and that this degradation process may also contribute to cytotoxicity. Nevertheless, several strategies have been developed to reduce or even prevent intracellular degradation, including polymeric coatings such as PEG [395], as well as inorganic coatings such as gold shells [396], which enhance nanoparticle stability and improve their safety profile. However, it should be emphasized that the overall safety profile of magnetic nanoparticles still depends on multiple factors, including their composition, coating, dose, and the target cell type.

Beyond cytocompatibility considerations, another limitation concerns the scalability of magnetically structured tissues. Magnetic manipulation relies on field gradients that decrease rapidly with distance, which can make it difficult to exert homogeneous forces throughout large tissue volumes [31]. Consequently, many magnetic tissue engineering demonstrations remain confined to millimeter-scale constructs or relatively thin tissues where magnetic forces remain effective [178]. Scaling these approaches toward clinically relevant dimensions may therefore require stronger or more complex magnetic systems, optimized nanoparticle loading, or hybrid fabrication strategies combining magnetic guidance with structural scaffolds. In addition, the spatial resolution and uniformity of magnetic actuation can be difficult to precisely control in complex 3D geometries, particularly when compared to fabrication-driven approaches such as bioprinting, which may limit the reproducibility of engineered anisotropic features. This challenge was observed when trying to assemble magnetic microtissues in space, where despite the complexity of the platform and the absence of gravity, there remained a lack of control on the final shape of the assembly [239]. Addressing these physical constraints will be essential to translate magnetic structuring strategies beyond proof-of-concept demonstrations.

Another important consideration is the potential for unintended side effects under dynamic magnetic fields, such as local heating under alternating magnetic fields or mechanical overstimulation of cells, which may affect tissue function if not carefully controlled. Furthermore, magnetic actuation typically requires the incorporation of exogenous materials, which distinguishes it from cell-driven or purely material-driven anisotropy and may introduce additional regulatory complexity. Despite these challenges, several factors support the translational potential of magnetic approaches. Iron oxide nanoparticles already have an established clinical track record as contrast agents for magnetic resonance imaging, including clinically approved formulations such as ferumoxytol [397], suggesting a relatively well-understood safety profile. Continued advances in nanoparticle engineering, magnetic field generation, and hybrid biofabrication strategies are therefore expected to improve both the safety and scalability of magnetic tissue engineering platforms. Future developments integrating magnetic actuation with other biofabrication strategies, such as 3D bioprinting or cell self-organization, may further help overcome current limitations by combining precise structural control with dynamic functionality. Together, these developments could enable the generation of anisotropic tissue constructs with dynamic and remotely controllable architectures, opening new avenues for the engineering of functional tissues with clinically relevant organization.

g. Positioning magnetic approaches within the landscape of anisotropic tissue engineering strategies

Despite their limitations, magnetic-based approaches offer a distinct paradigm for engineering anisotropic tissues compared to more conventional, non-magnetic strategies. While traditional methods primarily rely on material properties, fabrication processes, or intrinsic cellular behaviors to encode anisotropy, magnetic systems introduce the possibility of remote, dynamic, and reversible control over tissue organization. This fundamental difference has important implications in terms of spatial control, temporal tunability, and adaptability of engineered constructs, both before and after fabrication.

In most non-magnetic approaches, anisotropy is defined either by the structure of the scaffold or by the fabrication process itself. For instance, material-driven strategies such as electrospinning [127] or microfabricated substrates [133,134] rely on pre-defined topographical cues to guide cell alignment through contact guidance. Similarly, fabrication-based techniques including moulding, microfluidics, and bioprinting enable precise spatial patterning of cells and materials, but the resulting anisotropy is typically fixed once the construct is formed [170]. Although such approaches can achieve high levels of structural control and reproducibility, they often lack the ability to dynamically modulate tissue organization after fabrication. Moreover, the resolution of the aligned structures is usually limited by the resolution of the material itself or the fabrication process.

By contrast, magnetic approaches enable anisotropy to be induced, modified, or even reversed after the initial formation of the tissue. Alignment along magnetic field lines, spatial patterning through magnetic gradients [178], and the assembly of cellular building blocks can all be achieved without direct physical contact [31], allowing for the manipulation of tissues within enclosed or otherwise inaccessible environments. This remote controllability is particularly advantageous for dynamically regulating tissue architecture or applying mechanical stimuli in a temporally controlled manner [373,374], which is difficult to achieve with most conventional techniques. Importantly, once the magnetic platform is built, the application of magnetic forces is rapid and automatic, enabling alignment, assembly or patterning in a matter of minutes, whereas many fabrication platforms (e.g., extrusion bioprinting or MEW [238]) involve longer and stepwise processing times.

However, this added level of control comes with specific constraints. In contrast to scaffold-based approaches, where anisotropy is encoded in the material itself, magnetic strategies rely on the presence of magnetic elements, typically nanoparticles or magnetically responsive components, which introduces additional considerations related to biosafety, intracellular retention, and regulatory approval, as previously mentioned [392]. Moreover, while fabrication-based techniques such as bioprinting can achieve precise and reproducible spatial patterning at the macroscale, magnetic manipulation is often limited by the spatial resolution and penetration depth of magnetic field gradients, which can restrict the uniformity of anisotropy in larger constructs. Scaling up the size of engineered constructs therefore requires increasingly large [31,178] or convoluted magnetic set-ups [239], often involving very powerful magnets. Nonetheless, once again, the complexity of such platforms remains far lower than many non-magnetic platforms, such as 3D extrusion printing or laser-based polymerization or ablation systems.

From a structural standpoint, non-magnetic techniques generally offer stronger control over static architectural features, particularly at the macroscale, whereas magnetic approaches excel in enabling dynamic and adaptive anisotropy, including the ability to apply forces, induce deformation, or reorganize cellular structures over time. These differences highlight that magnetic and non-magnetic strategies are not directly interchangeable but rather address complementary aspects of anisotropic tissue engineering.

Overall, magnetic approaches extend the design space of anisotropic tissue engineering by introducing a level of temporal and remote control that is not readily accessible with conventional methods. When considered alongside established techniques, they provide a distinct set of capabilities that can be leveraged depending on the desired balance between structural precision, dynamic regulation, and translational constraints. Future strategies will likely benefit from hybridization, combining the spatial accuracy of fabrication-based methods with the dynamic control enabled by magnetic actuation.

## 6 Future perspectives for engineering anisotropic tissues

a. Hybrid and dynamic biofabrication strategies for anisotropic tissues

The integration of magnetic actuation with established biofabrication techniques represents a promising strategy to combine precise structural design with dynamic and remote control of tissue organization. Established approaches such as 3D bioprinting, microfluidics, and scaffold-based fabrication provide precise spatial control over geometry and composition, yet they remain largely limited to static architectures. In contrast, magnetic systems offer remote, non-contact control and the ability to dynamically modulate tissue organization before and after fabrication, representing a promising direction for overcoming current limitations in anisotropic tissue engineering.

Indeed, fabrication-based techniques often struggle to generate a 3D distribution of anisotropic microfeatures. Including magnetic elements within a gel to align magnetically during the fabrication process could be a way to engineer multi-level anisotropy [151,216]. It can also be leveraged to reinforce existing effects, as was done by magnetically aligning a gel as it was extruded to enhance shear-induced alignment [174]. Even in melt-electrowriting, where it is possible to achieve very fine anisotropic architectures, aligning magnetic cells could enable the combination of an anisotropic cell organization with the material anisotropy. More generally, this approach makes it possible to organize one magnetic material and one non-magnetic material in two different ways. For instance, a gel can be moulded with in it one cell type that can align magnetically while the other, non-magnetic, is entirely dependent on the general anisotropy of the gel [31]. The possibilities to magnetically pattern already anisotropic scaffolds with cells, or to precisely position and assemble spheroids remotely within a bioprinted construct [286,292], offer additional layers of structural control with multiple anisotropic features at once. However, integrating magnetic components without compromising printability or material properties would likely be a key challenge of such approach.

The potential for remote control is also particularly interesting for actuation of a tissue after its fabrication, whether it was engineered magnetically or not. For instance, combining magnetic actuation with 3D bioprinting could enable the fabrication of constructs with predefined geometry that can subsequently be reconfigured, such as a 3D printed flower-shaped construct able to move with a crab-like motion via alternating magnetic forces [386]. More generally, any scaffold with magnetic components in it, can first be given an anisotropic structure with a non-magnetic approach, and later be remodeled or stimulated through field-induced alignment, deformation, stiffening or even heating [370]. Having only one portion of the gel magnetic is another way to apply directed forces for stimulation, without necessarily including magnetic elements throughout the construct [363]. Microfluidic systems could be coupled with magnetic forces to guide cell organization during or after flow-based patterning, enabling targeted spatial control within complex geometries. Such hybrid strategies would allow the decoupling of structural definition from functional maturation, enabling anisotropy to be progressively introduced, tuned, or even reprogrammed over time.

Beyond their integration with fabrication techniques, magnetic forces also offer opportunities to synergize with intrinsic cell-driven processes. While these mechanisms can spontaneously generate anisotropy, they are often difficult to control spatially and temporally. Magnetic actuation can provide an additional level of regulation by guiding initial cell orientation, applying directional mechanical cues [387], or dynamically modulating the microenvironment during tissue maturation. For instance, magnetic alignment of cells or matrix components may serve as a template that is subsequently reinforced by cell contractility and matrix deposition [178]. Moreover, cyclic or time-varying magnetic stimulation could influence processes such as matrix remodeling or collective cell migration, enabling the progressive emergence of functional anisotropy. Conversely, magnetic forces could also potentially counteract certain forms of cellular reorganization [364], for instance if surface tension sorting leads to undesired cell arrangements. They could also be leveraged to probe these intrinsic cellular mechanisms, providing quantitative data on the forces at play and what is needed to balance them. That said, the complexity of these interactions makes predictive control difficult, and remains an open challenge. Nonetheless, such synergistic approaches highlight the potential of combining external physical control with intrinsic biological self-organization to achieve more robust, adaptive, and physiologically relevant anisotropic tissues.

More broadly, these approaches pave the way toward four-dimensional (4D) tissue engineering, where tissue architecture evolves in response to external stimuli. Unlike static scaffolds, magnetically responsive systems can generate time-dependent mechanical cues, guide cell migration and reorganization. This dynamic control is particularly relevant for mimicking developmental processes or physiological remodeling, where anisotropy is continuously adapted rather than fixed. Future works may therefore focus on programmable materials and feedback-controlled magnetic systems capable of spatiotemporal regulation of tissue organization and function. Achieving this level of control will likely require integrating sensing, modeling, and actuation within unified platforms.

b. From increasing multiscale control to improving quantitative standardization of anisotropy

A central challenge in anisotropic tissue engineering lies in reproducing the hierarchical organization of native tissues across multiple length scales. As exemplified with muscle tissue, where alignment spans from the whole organ to every single cell and myofibril, native anisotropy emerges from coordinated structural micro- and sometimes even nano-features to macroscale geometry, which cannot typically be achieved using a single fabrication strategy. The few cases where both macroscopic and microscopic anisotropy are controlled usually combine bioprinting or moulding with external fields, such as temperature gradients to engineer pores or electric forces for layers [371]. Magnetic approaches offer unique opportunities in this context, as they can act simultaneously at the cellular and tissue levels, enabling alignment, assembly, and mechanical stimulation within a unified framework. When combined with other physical cues such as mechanical loading, electrical stimulation, or fluid flow, these systems may enable multiphysics control over tissue organization, more closely recapitulating the complex environments that govern anisotropy *in vivo* across scales. Achieving consistent coordination across these scales remains a major unresolved challenge.

The ability to design increasingly complex anisotropic systems also highlights the need for robust and standardized methods to quantify these features. While numerous studies report alignment or directional properties, the metrics used are often inconsistent, making cross-study comparisons difficult. Establishing quantitative benchmarks, such as alignment indices, orientation distributions [178], directional modulus ratios [46,47], or anisotropic transport coefficients, will be essential to objectively evaluate and compare different strategies. In parallel, the development of standardized experimental protocols and reporting guidelines would improve reproducibility and facilitate the identification of optimal approaches for specific applications. Importantly, the selection of relevant metrics should be adapted to the intended application of the engineered construct. *In vitro* models, such as organs-on-chip, would most likely focus first on recapitulating architectural anisotropy to gain a better understanding on cellular mechanisms, whereas for implantation, functional anisotropy is often more critical. Similarly, the relevance of local versus macroscopic measurements depends on tissue scale and function. In the case of engineered skeletal muscle constructs, macroscopic functional anisotropy is usually quantified by measuring the specific force normalized per surface area [165], while the anisotropy of the microscopic internal organization is usually evaluated via cellular alignment [153]. However, only few approaches have quantified local stresses in 3D so far [178]. Ultimately, combining multiscale standardized frameworks with application-specific metrics should enable the development of engineered tissues that more faithfully replicate the native hierarchical anisotropic properties.

These efforts would also help clarify the relative contributions of material properties, fabrication methods, and cell-driven processes in generating anisotropy. By providing a common framework for evaluation, they would enable a more rigorous comparison between strategies and support the rational design of anisotropic tissues with predictable structural and functional outcomes, which is essential for translational applications. Such standardization will be particularly important as hybrid and multiphysics approaches continue to increase system complexity.

c. Toward adaptative anisotropic systems for *in vivo* integration

The translation of anisotropic tissue engineering strategies toward *in vivo* and clinical applications represents a critical next step for the field. Beyond the technical limitations discussed previously, achieving functional integration with host tissues remains a major challenge.

In this context, magnetic tissues may offer unique opportunities to improve implantation of engineered tissues and their integration. Their magnetic properties could be leveraged to enhance precision and reduce invasiveness during operations. Indeed, cellular patches can be engineered in defined geometries matching the defect site, and magnetic trapping or actuation can facilitate positioning. Tissues magnetically bioprinted out of cells labeled with iron oxide have for instance been shaped into clamp-like structures capable of attaching to magnetic supports [178]. Similarly, magnetic stents could be used to guide and then anchor the tissues during implantation. Such possibilities are supported by previous approaches aiming to guide magnetic vectors [398] and even cells *in vivo* [399], further reinforcing the possibility of guiding magnetic grafts during implantation. Moreover, magnetic forces could be leveraged to better bind constructs to the implantation site [350]. For instance, in the case of cartilage repair, cells or tissues implanted within a defect to fill it tend to diffuse after operation, and even implanting a printed scaffold requires a perfect press fit into a hole in the subchondral bone to prevent detachment. Remotely applying magnetic pressure on such implanted tissues could improve integration and reduce the risk of displacement, although this requires careful control of applied forces. Post-implantation, the magnetic properties could also enable ongoing guidance, stimulation [216,220], and monitoring via MRI [26], providing a level of control not achievable with most conventional approaches.

In particular, engineered constructs must support vascularization, innervation, and mechanical coupling while maintaining their anisotropic organization in a dynamic physiological environment. Indeed, anisotropy in native tissues is not static but continuously remodeled. Preserving or guiding this organization after implantation will be essential for long-term functionality and magnetic approaches could actively modulate tissue organization after implantation. Advances in magnetic field generation, including miniaturized, wearable, or implantable systems, may enable remote and non-invasive control of tissue structure and mechanics *in vivo*. In parallel, hybrid strategies combining magnetic actuation with scaffold-based or bioprinted constructs could improve structural stability while retaining dynamic tunability [174,370]. Such approaches may help bridge the gap between precisely engineered architectures and the adaptive behavior of living tissues, although their effectiveness in deep tissues will depend on achievable field strength and spatial precision.

Looking forward, the convergence of anisotropic tissue engineering with biohybrid and adaptive systems may further expand the field. By integrating cells capable of sensing and responding to their environment with externally controllable magnetic cues, it may become possible to engineer tissues that dynamically adjust their anisotropy in response to physiological signals. These adaptive systems could enable real-time regulation of structure and function, moving beyond static constructs toward living materials with self-regulating properties. Together, these developments highlight a shift from the fabrication of anisotropic structures to the design of dynamic, functional tissues capable of long-term integration and adaptation *in vivo*.

## CRediT authorship contribution statement

**Noam Demri:** Conceptualization, Funding acquisition, Investigation, Visualization, Writing – original draft, Writing – review & editing. **Stéphanie Descroix:** Conceptualization, Funding acquisition, Supervision, Writing – review & editing. **Claire Wilhelm:** Conceptualization, Funding acquisition, Project administration, Supervision, Validation, Writing – review & editing.

## Declaration of competing interest

The authors declare the following financial interests/personal relationships which may be considered as potential competing interests: Claire Wilhelm reports financial support was provided by French National Research Agency. Claire Wilhelm reports financial support was provided by Pierre-Gilles de Gennes Institute. Claire Wilhelm reports financial support and administrative support were provided by Institut Curie. If there are other authors, they declare that they have no known competing financial interests or personal relationships that could have appeared to influence the work reported in this paper.

## Data Availability

No data was used for the research described in the article.
